# Supplementary material for: Quantifying portable genetic effects and improving cross-ancestry genetic prediction with GWAS summary statistics
Source: Nat Commun. 2023 Feb 14;14:832. doi: 10.1038/s41467-023-36544-7 (PMC9929290; doi:10.1038/s41467-023-36544-7)
Supplement: Supplementary file 1 — Supplementary Information [file 41467_2023_36544_MOESM1_ESM.pdf]

Supplementary Information

Quantifying portable genetic effects and improving cross-ancestry genetic prediction with GWAS summary statistics

Jiacheng Miao[1,*], Hanmin Guo[2,*], Gefei Song[1], Zijie Zhao[1], Lin Hou[2,3,†,#], Qiongshi Lu[1,4,5,†,#]

[1] Department of Biostatistics and Medical Informatics, University of Wisconsin–Madison, WI, USA 53706
[2] Center for Statistical Science, Department of Industrial Engineering, Tsinghua University, Beijing, China 100084
[3] MOE Key Laboratory of Bioinformatics, School of Life Sciences, Tsinghua University, Beijing, China 100084
[4] Department of Statistics, University of Wisconsin–Madison, Madison, WI, USA 53706
[5] Center for Demography of Health and Aging, University of Wisconsin–Madison, Madison, WI, USA 53706

* These authors contributed equally to this work: Jiacheng Miao, Hanmin Guo
† These authors jointly supervised this work: Lin Hou, Qiongshi Lu

# To whom correspondence should be addressed:
Dr. Qiongshi Lu (qlu@biostat.wisc.edu) and Dr. Lin Hou (houl@tsinghua.edu.cn)

This PDF file includes:

Supplementary Fig. 1-32

Supplementary Methods

Supplementary References

Supplementary Figures

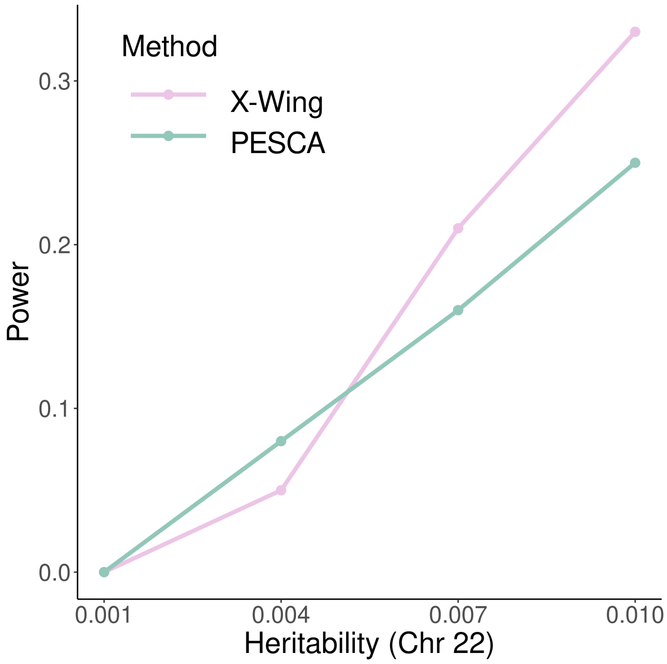

Supplementary Figure 1. Statistical power in simulations under LDAK model in which SNP heritability is dependent on LD and MAF.

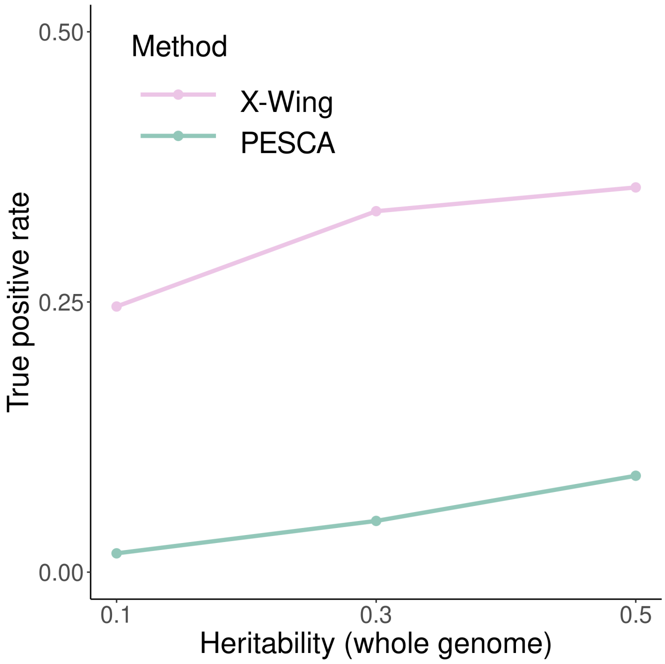

Supplementary Figure 2. True positive rates in simulations under heritability enrichment model for whole genome SNPs.

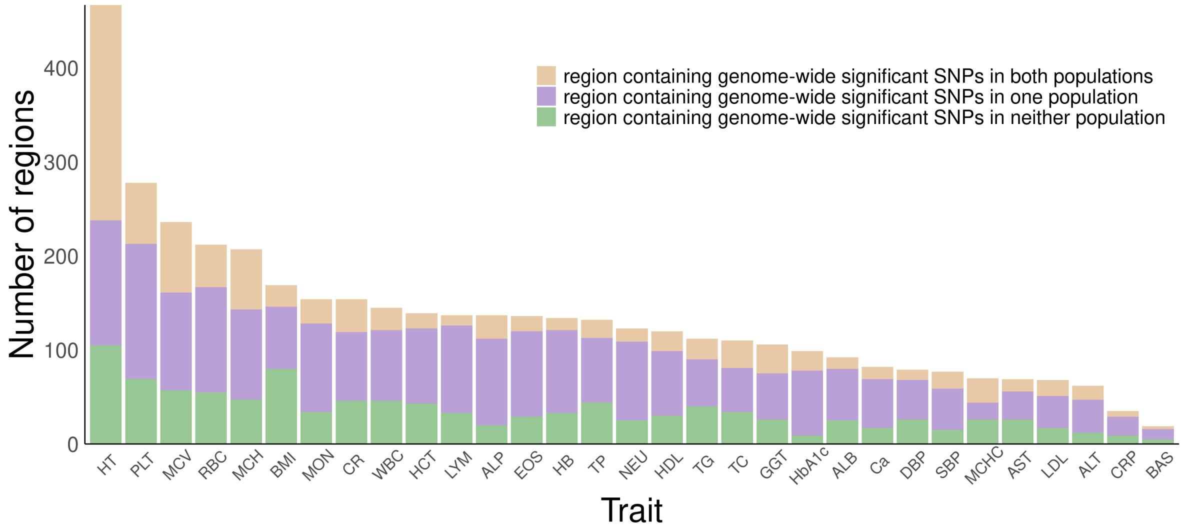

Supplementary Figure 3. Number of regions with significant local genetic correlations between Europeans and East Asians in 31 complex traits. Three bars denote regions containing genome-wide significant SNPs in both populations (brown), in only one population (purple), and in neither population (green).

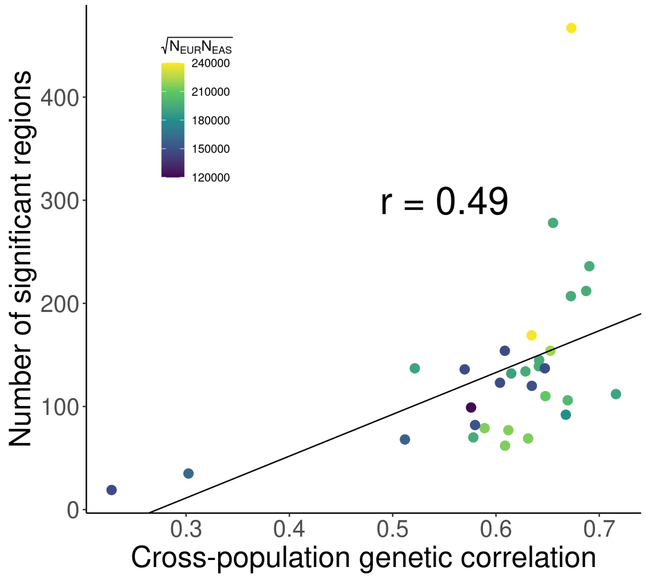

Supplementary Figure 4. Number of X-Wing-identified regions is proportional to cross-population genetic correlation. GWAS sample size are indicated by the color of each data point.

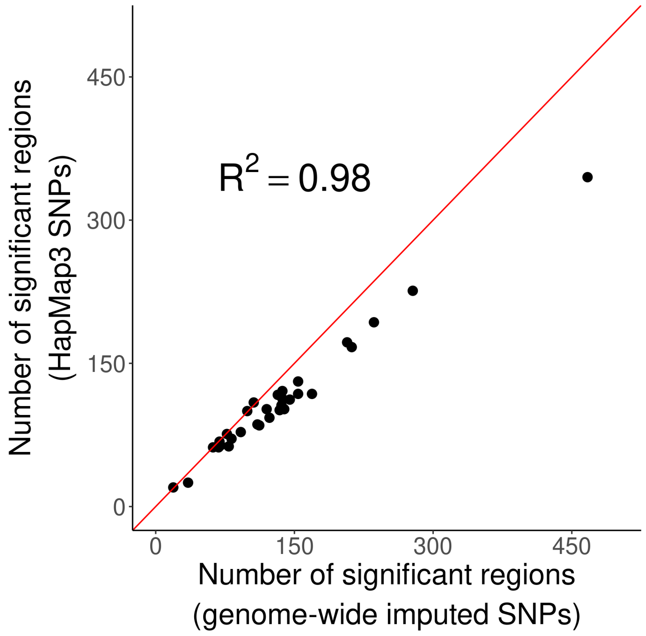

Supplementary Figure 5. Number of significant regions identified by X-Wing using genome-wide imputed SNPs and HapMap3 SNPs for 31 complex traits.

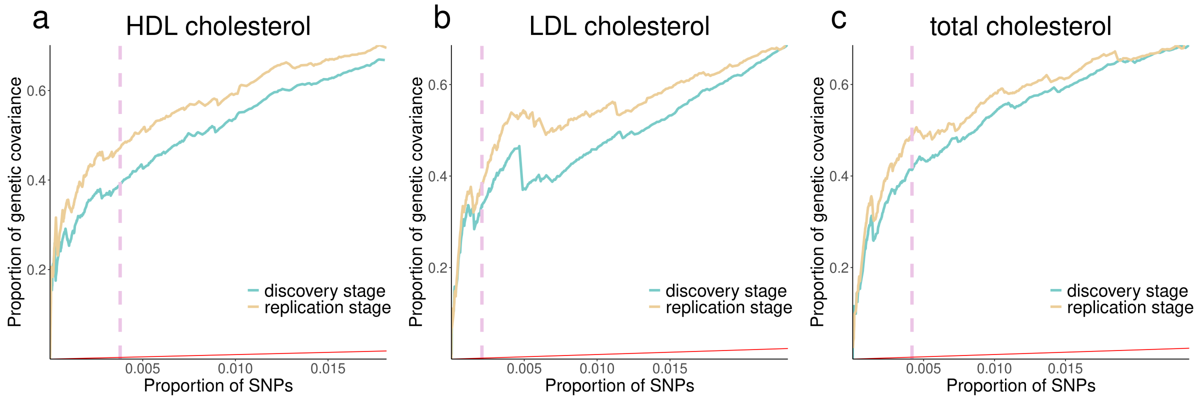

Supplementary Figure 6. Cumulative proportion of genetic covariance explained by regions identified in the discovery stage for HDL cholesterol, LDL cholesterol, and total cholesterol. Panels **a, b,** and **c** are the results for HDL cholesterol, LDL cholesterol, and total cholesterol, respectively. Pink dashed line indicates FDR cutoff of 0.05. Red line indicates diagonal line of y=x.

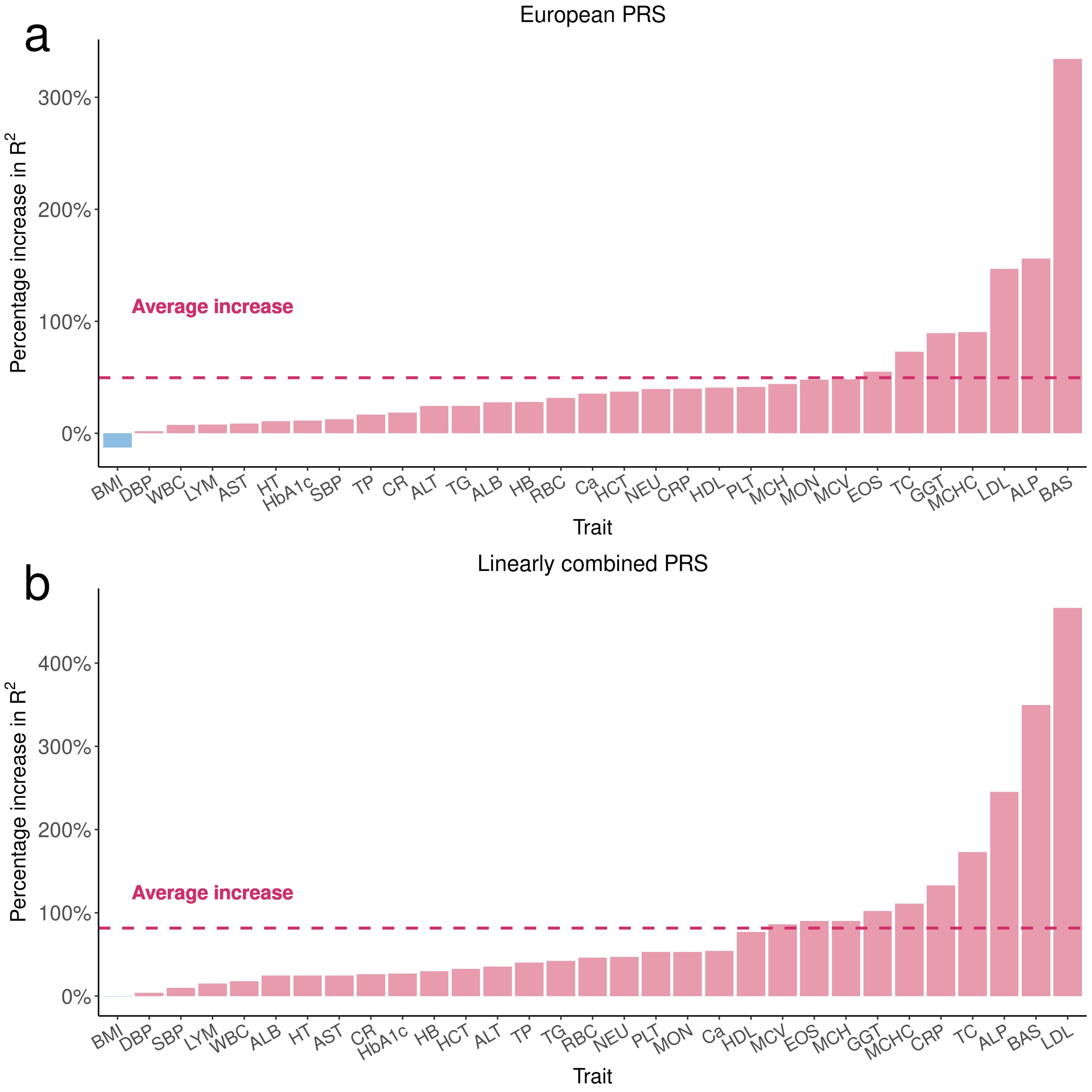

Supplementary Figure 7. Comparison of the prediction accuracy between X-Wing and XPASS PRS for 31 traits in East Asian sample. Panels **a** and **b** illustrate the percentage increase in R^2 of X-Wing European and linearly combined PRS over XPASS, respectively. The dashed line represents the average increase.

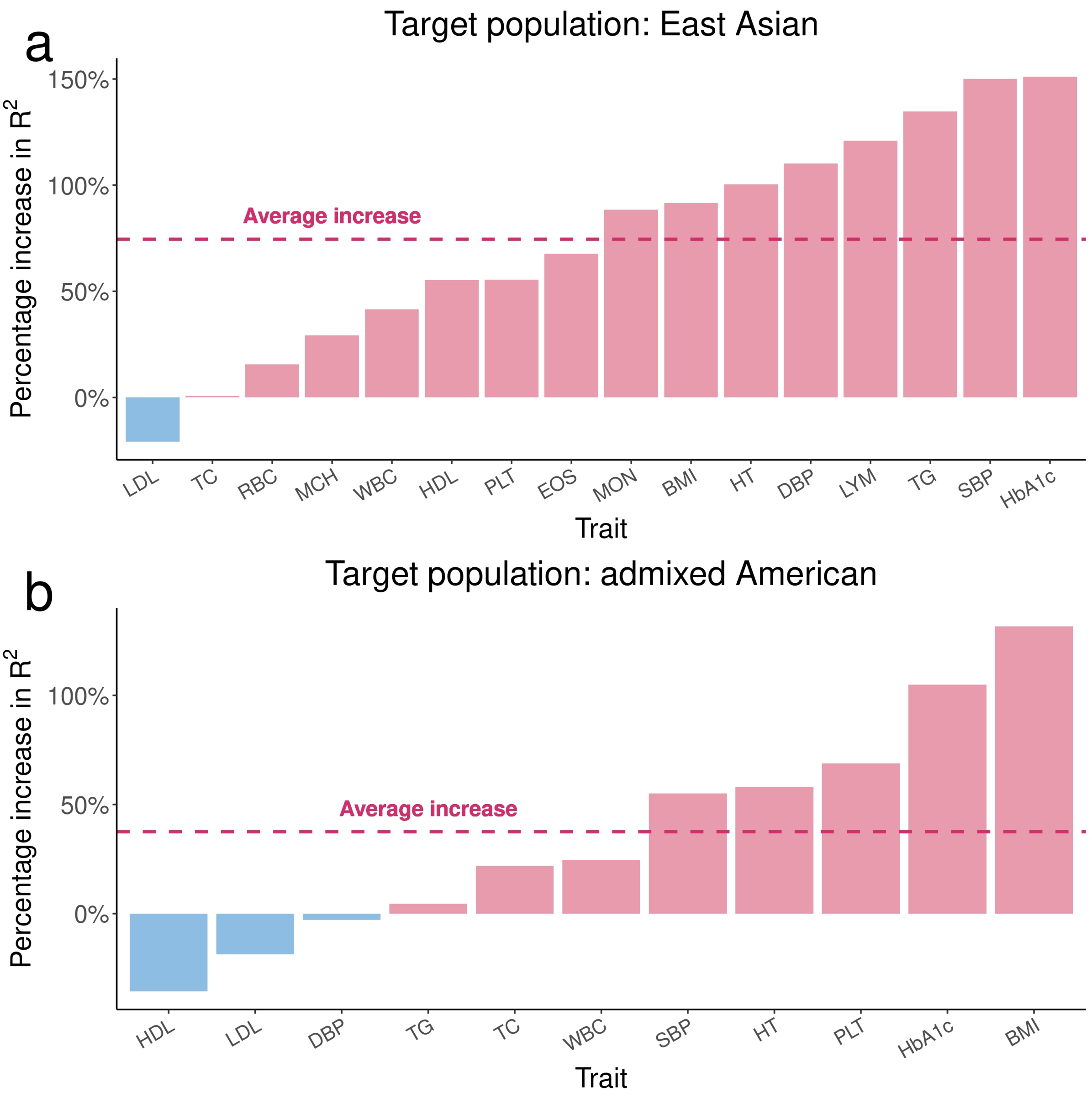

Supplementary Figure 8. Comparison of the prediction accuracy between X-Wing and PolyFun-pred European PRS. Panels **a** and **b** show the percentage increase in R^2 of X-Wing European PRS over PolyFun-pred for 16 traits in East Asians and 11 traits in admixed Americans, respectively.

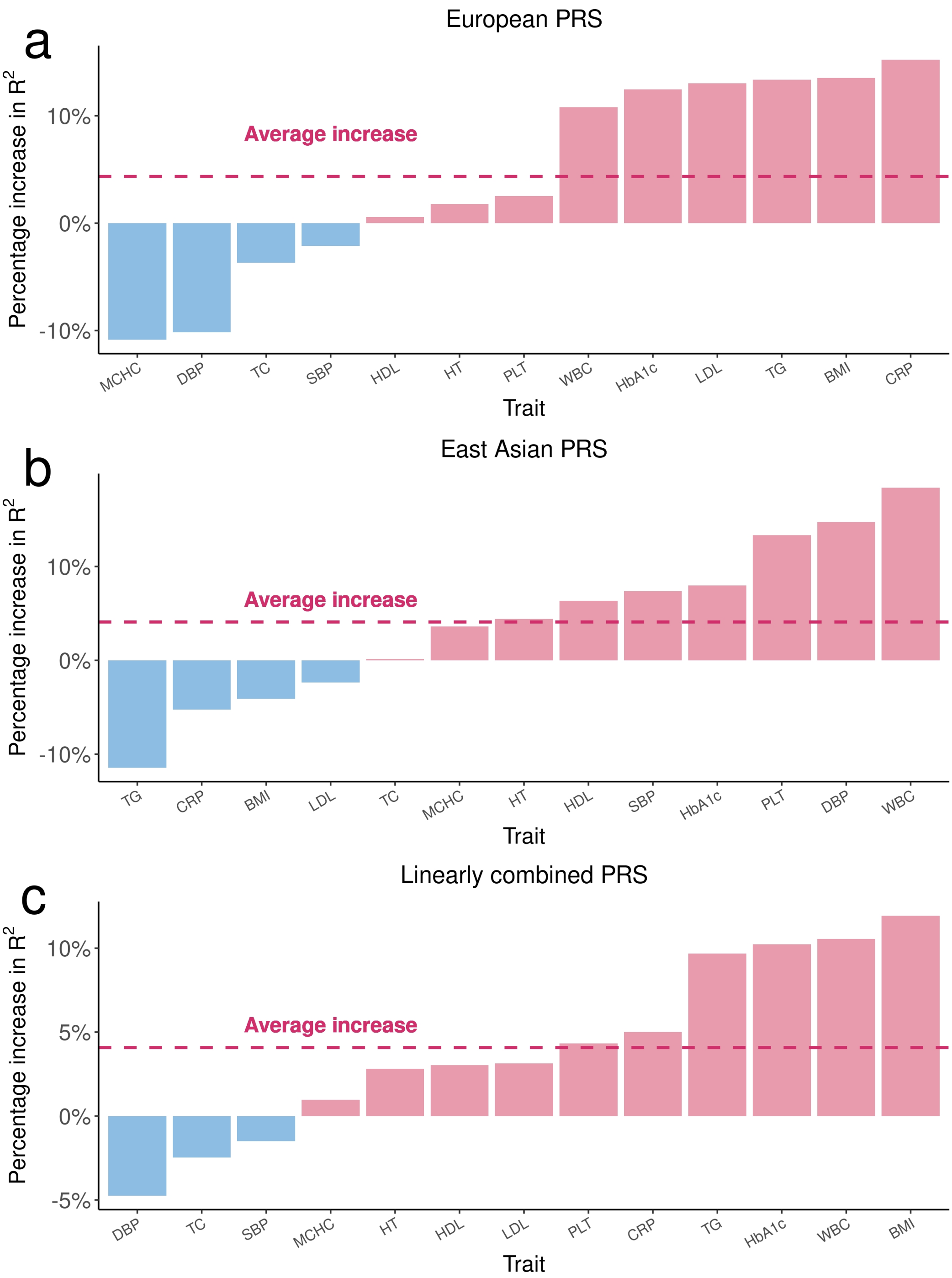

Supplementary Figure 9. Comparison of the prediction accuracy between X-Wing and PRS-CSx PRS for 13 traits in admixed American sample. Panels **a, b,** and **c** illustrate the percentage increase in R^2 of X-Wing European, East Asian, and linearly combined PRS over PRS-CSx. respectively. The dashed line represents the average increase.

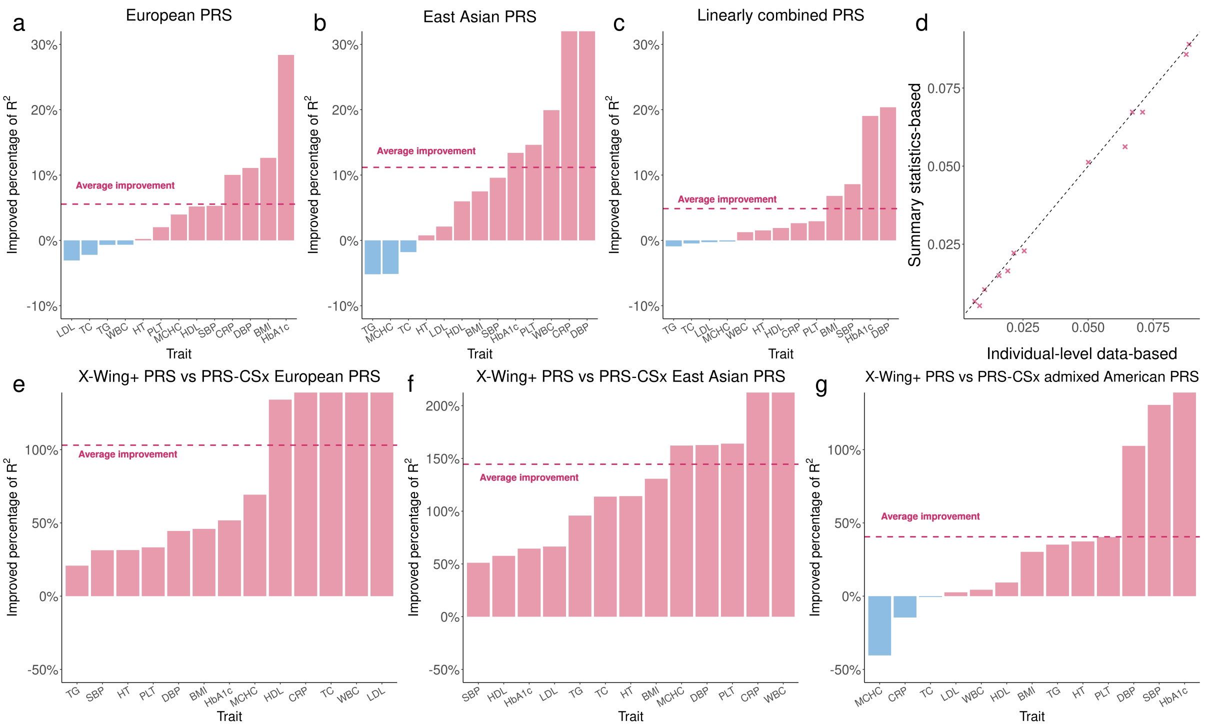

Supplementary Figure 10. Performance of X-Wing for 13 traits in African ancestry samples. Panel **a, b**, and **c** show the percentage increase in R² of X-Wing over PRS-CSx for European, East Asian, and linearly combined PRS. Panel **d** compares the R² for linearly combined PRS with mixing weights obtained using GWAS summary statistics and individual-level data. Panel **e, f**, and **g** show the percentage increase in R² of X-Wing PRS over PRS-CSx using only GWAS summary statistics. X-Wing+ PRS is the linearly combined X-Wing PRS using weights estimated using GWAS summary statistics. PRS-CSx PRS is calculated based on posterior mean effects of European, East Asian, and admixed American population, respectively. The dashed line represents the average increase.

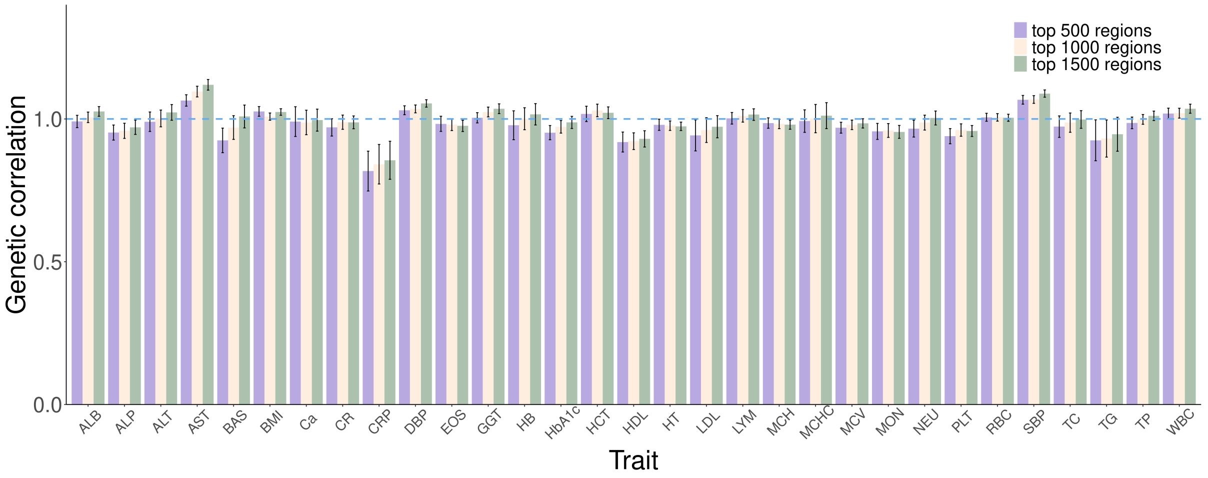

Supplementary Figure 11. Bar plot shows the cross-population genetic correlation estimates and standard errors using varying numbers of top annotated regions. The error bar represents ± 1 standard errors of the estimates. The centre for the error bars represents the point estimates for genetic correlation. A list of trait acronyms and the corresponding sample size can be found in **Supplementary Table 7.**

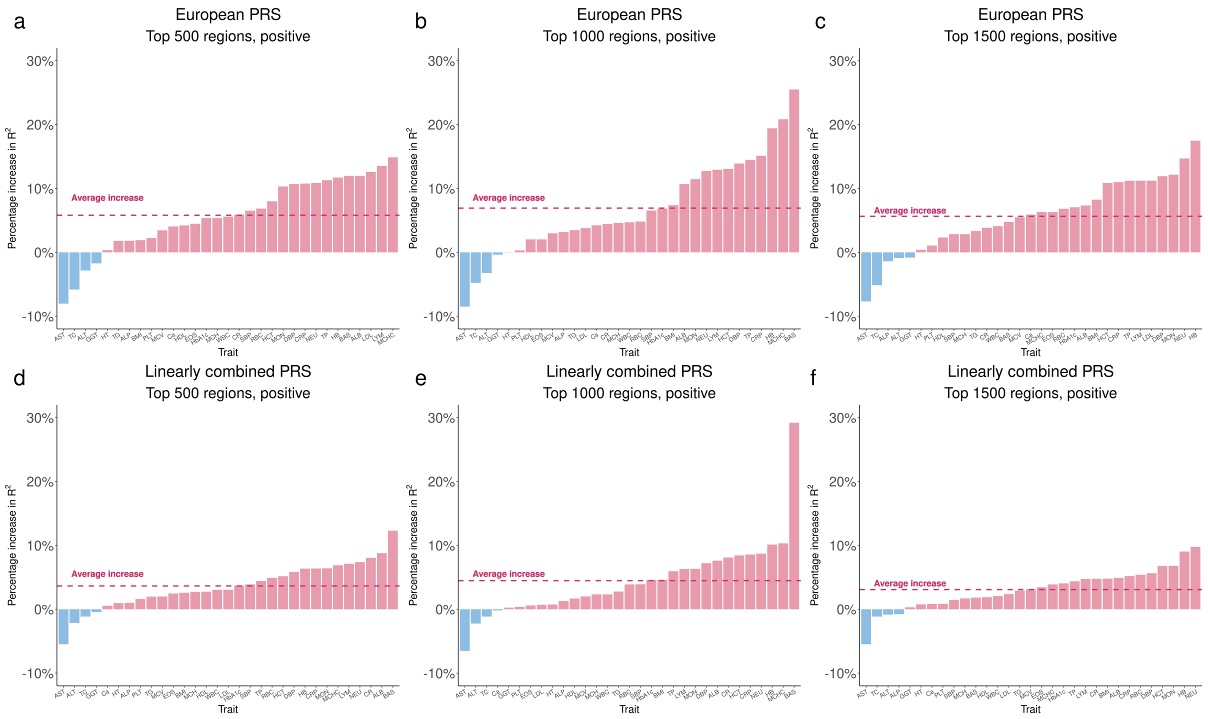

Supplementary Figure 12. Comparison of the prediction accuracy between X-Wing and PRS-CSx PRS for 31 traits in East Asians with varying numbers of top regions. Panel **a, b**, and **c** show the percentage increase in R^2 of X-Wing European PRS over PRS-CSx using 500, 1000, and 1500 positive regions as annotation. Panel **d, e**, and **f** are the results for linearly combined PRS. The dashed line represents the average increase.

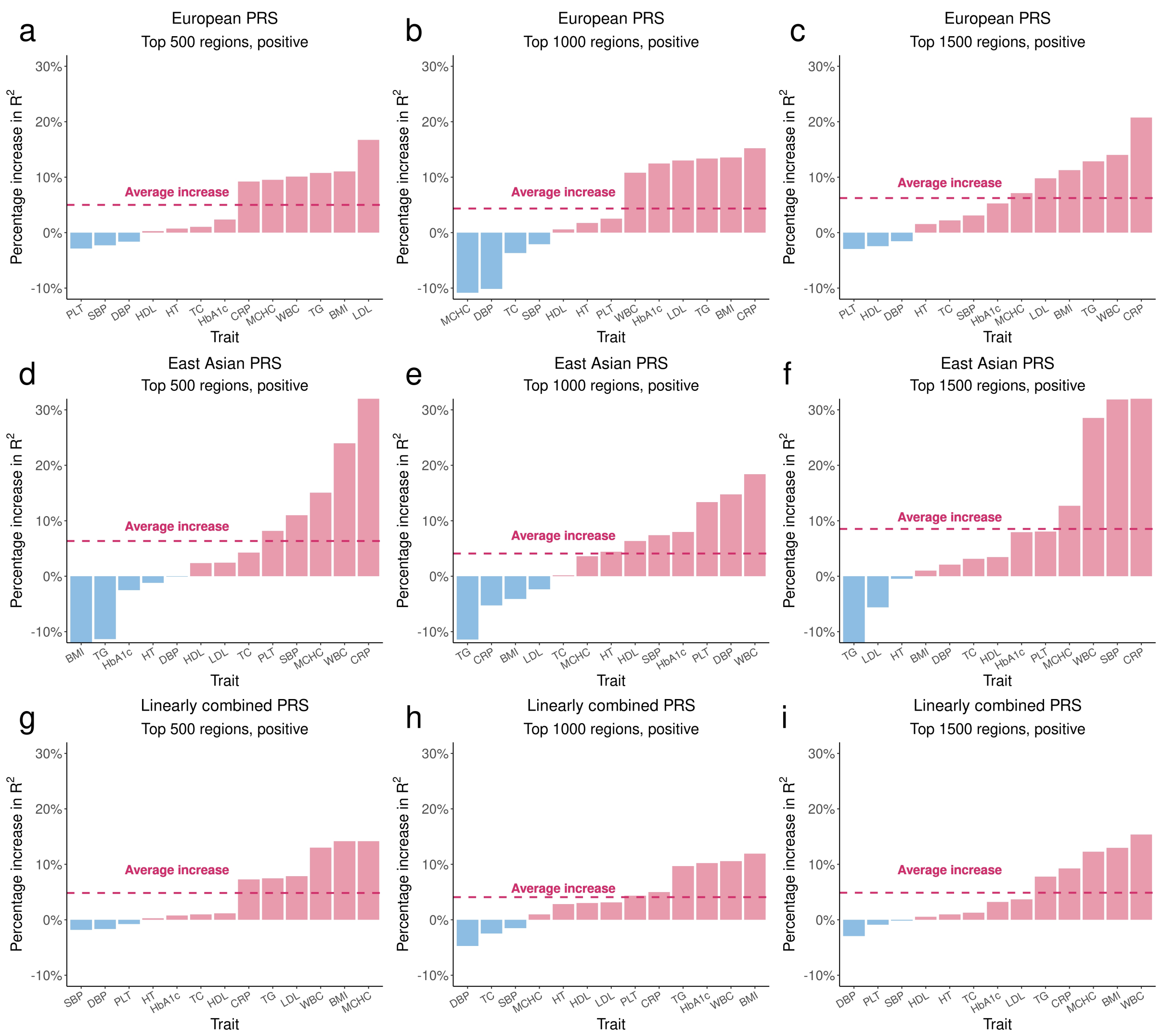

Supplementary Figure 13. Comparison of the prediction accuracy between X-Wing and PRS-CSx PRS for 13 traits in admixed Americans with varying numbers of top regions. Panel **a, d**, and **g** show the percentage increase in R^2 of European PRS from X-Wing over PRS-CSx using 500, 1000, and 1500 positive regions as annotation. Panel **b, e**, and **h** are the results for East Asian PRS. Panel **c, f**, and **i** represent the results for linearly combined PRS. The dashed line represents the average increase.

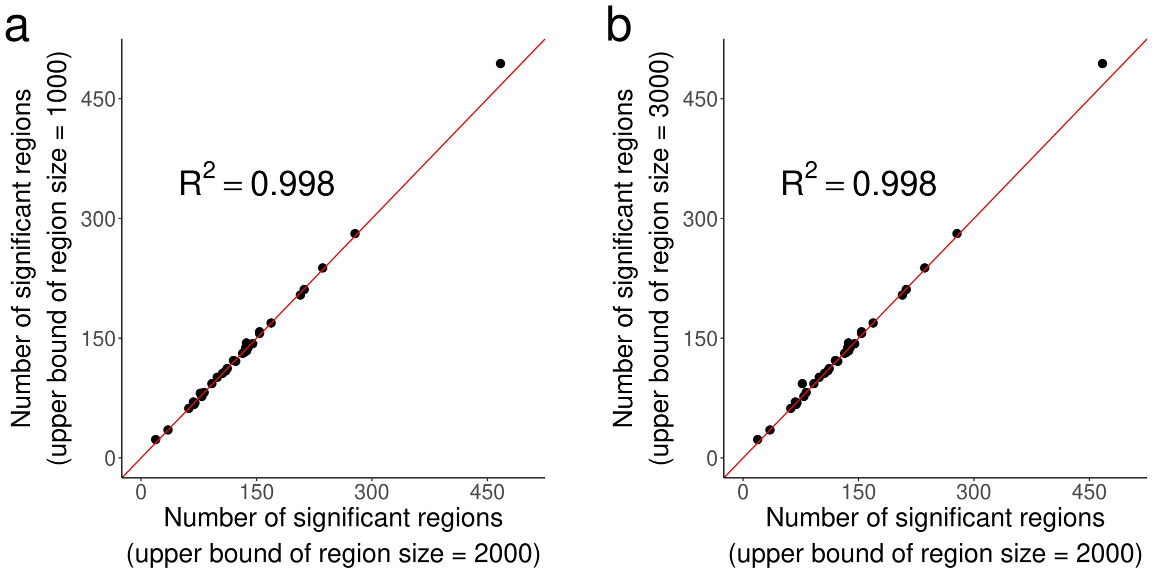

Supplementary Figure 14. Number of significant regions identified by X-Wing for 31 complex traits with different upper bound of region size. Panels **a** and **b** are the results for upper bound of region size = 1000 and 3000, respectively.

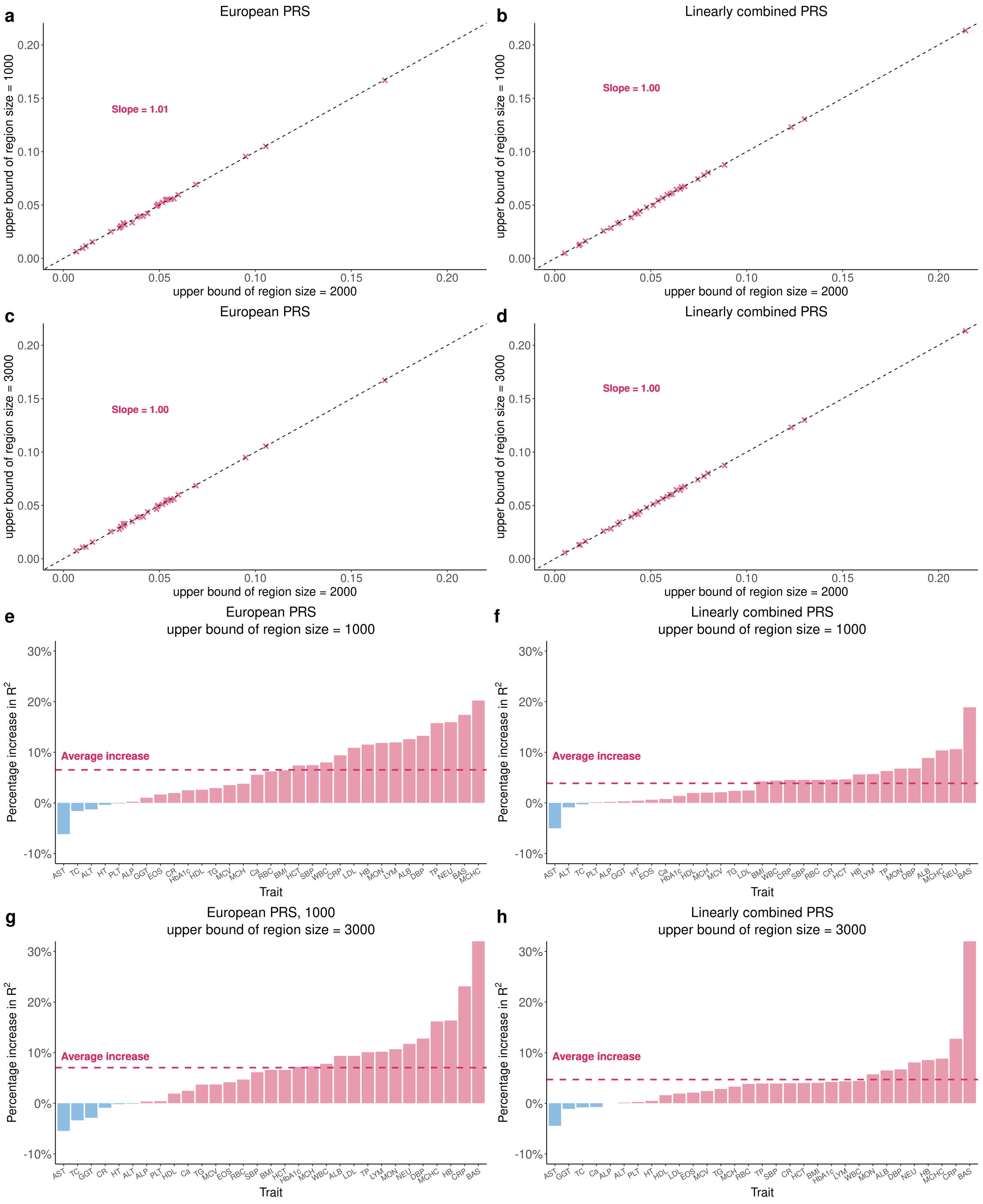

Supplementary Figure 15. Comparison of the prediction accuracy between X-Wing and PRS-CSx PRS for 31 traits in East Asians with varying upper bound of region size. Panel **a-d:** X-Wing PRS with upper bound of region size=1000. Panel **e-h:** X-Wing PRS with upper bound of region size=3000. The baseline in panel **e-h** is the PRS-CSx. The P-value of two-sided Wilcoxon signed-rank test for analysis in each panel are (**a**)0.92; (**b**)0.90; (**c**)0.45; (**d**)0.26; (**e**)2.6e-6; (**f**)5.0e-7; (**g**)4.0e-6; (**h**)5.0e-7.

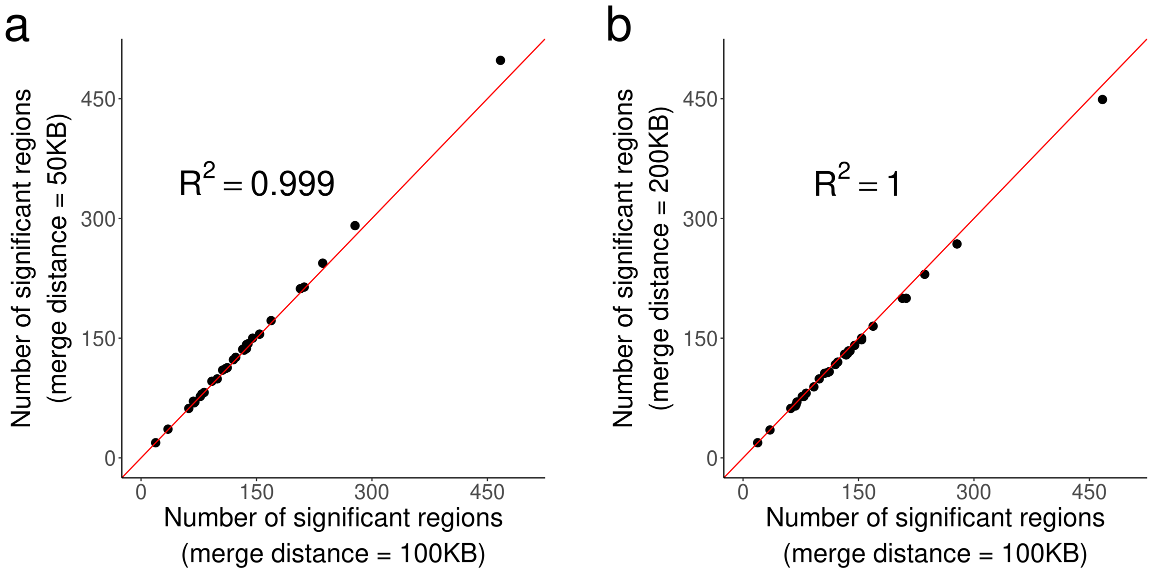

Supplementary Figure 16. Number of significant regions identified by X-Wing for 31 complex traits with different merge distance. Panels **a** and **b** are the results for merging distance = 50kb and 200 kb, respectively.

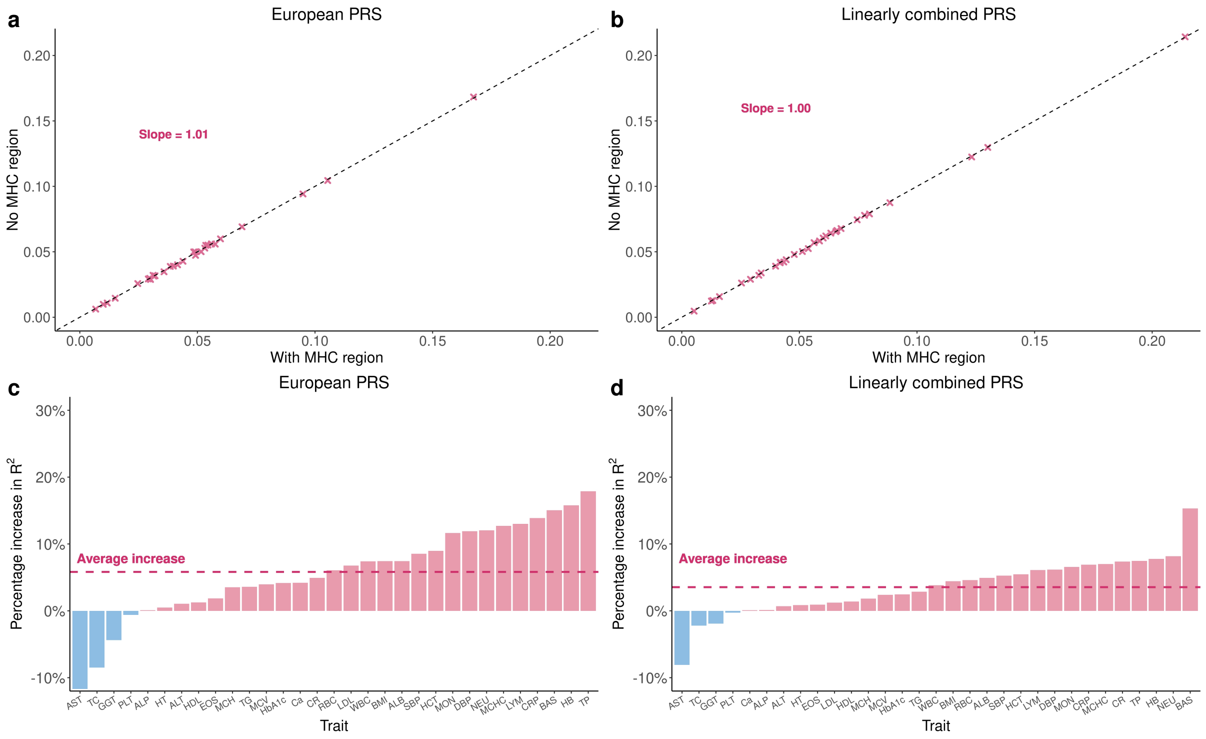

Supplementary Figure 17. Impact of exlcuding MHC region in identifying regions with local genetic correlation on PRS prediction accuracy for 31 traits in East Asians. (**a**) X-Wing (annotation that excludes MHC region)vs X-Wing (annotation that includes MHC region) European PRS: ($P_{wilcoxon} = 0.22$). (**b**) X-Wing (annotation that excludes MHC region)+ vs X-Wing (annotation that includes MHC region) linearly combined PRS: ($P_{wilcoxon} = 0.09$). (**c**) X-Wing (annotation that excludes MHC region) vs PRS-CSx European PRS ($P_{wilcoxon} = 7.0e-05$). (**d**) X-Wing (annotation that excludes MHC region) vs PRS-CSx linearly combined PRS ($P_{wilcoxon} = 1.2e-05$). All annotations are based on top 1000 positive regions. The P-value is calculated using two-sided Wilcoxon signed-rank test.

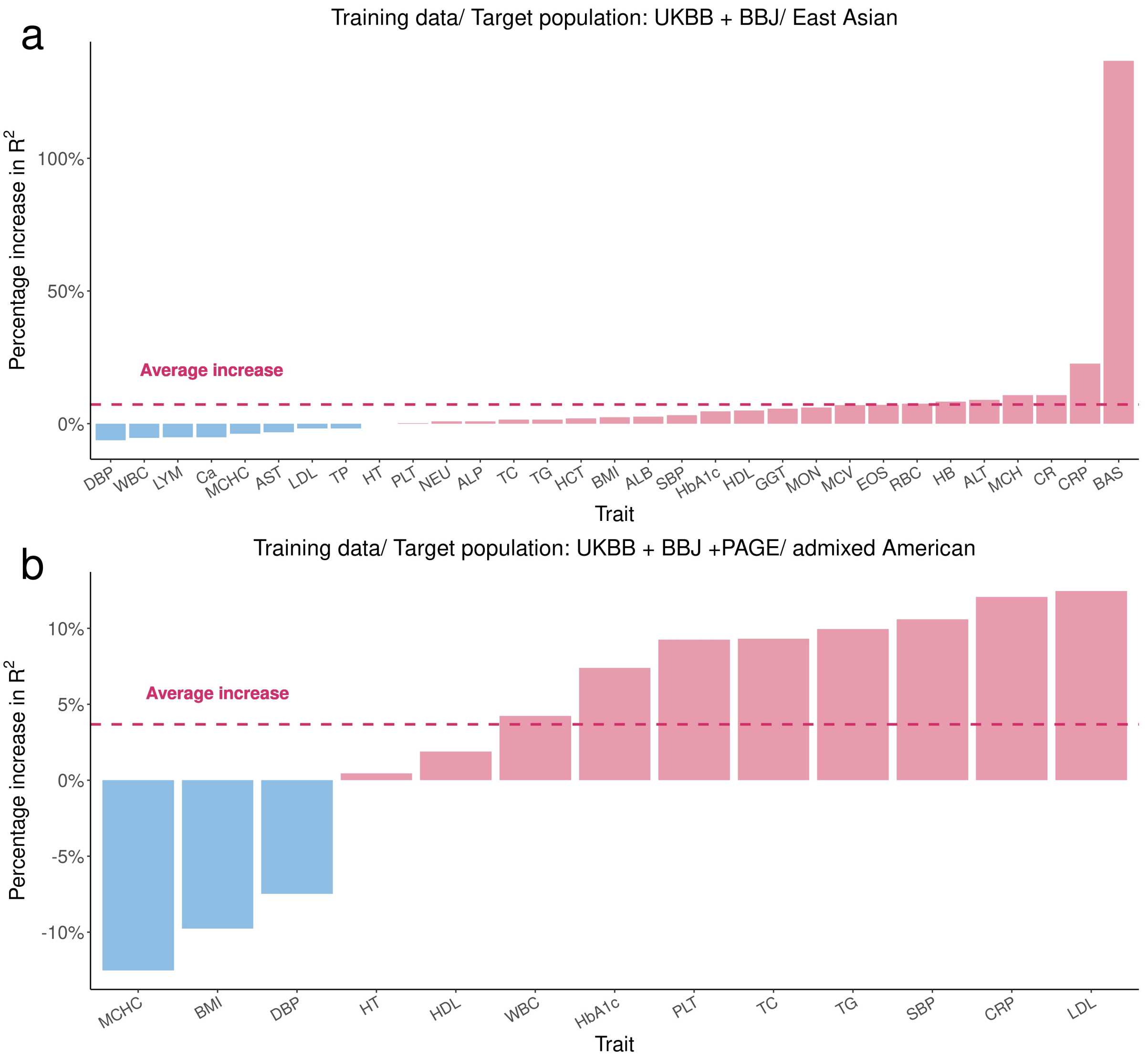

Supplementary Figure 18. Comparison of the prediction accuracy between X-Wing and PRS-CSx PRS when using tuning parameter to select global shrinkage parameter. The percentage increase in R^2 of linearly combined PRS from X-Wing over PRS-CSx is shown in **(a)** for 31 traits in East Asians and **(b)** for 13 traits in admixed Americans.

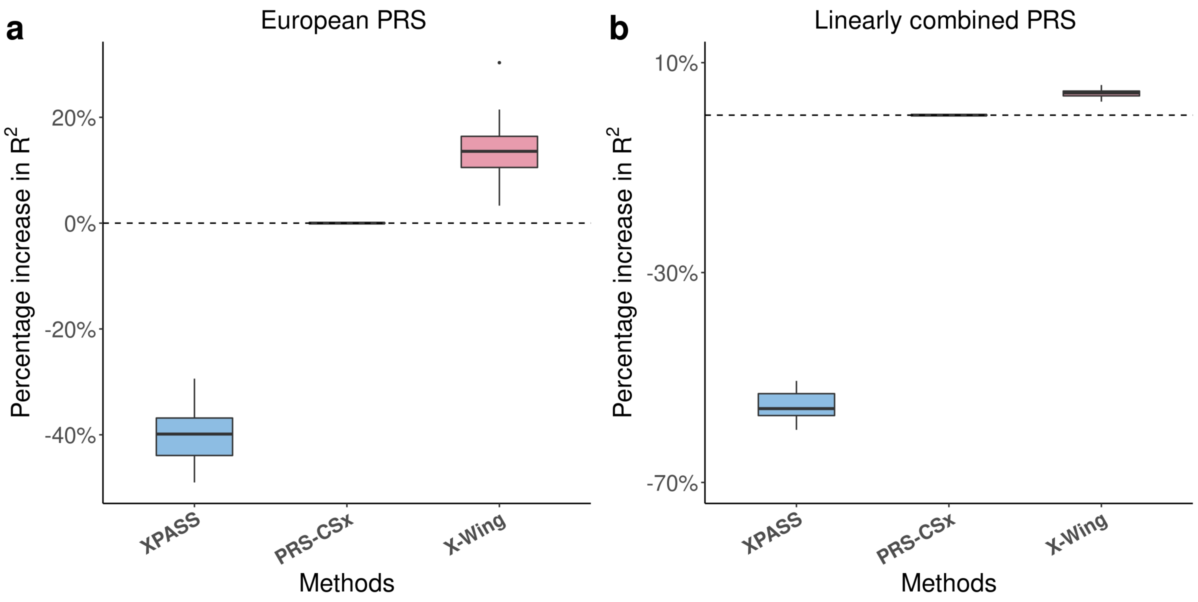

Supplementary Figure 19. Predictive accuracy (measured by R^2) of X-Wing, PRS-CSx, and XPASS in simulations. The boxes represent the percentages relative increase of R^2 over PRS-CSx across 20 simulation replicates. The PRS is **(a)** European PRS **(b)** Linearly combined PRS. In these boxplots, the center line, box limits and whiskers denote the median, upper and lower quartiles, and 1.5 × interquartile range, respectively.

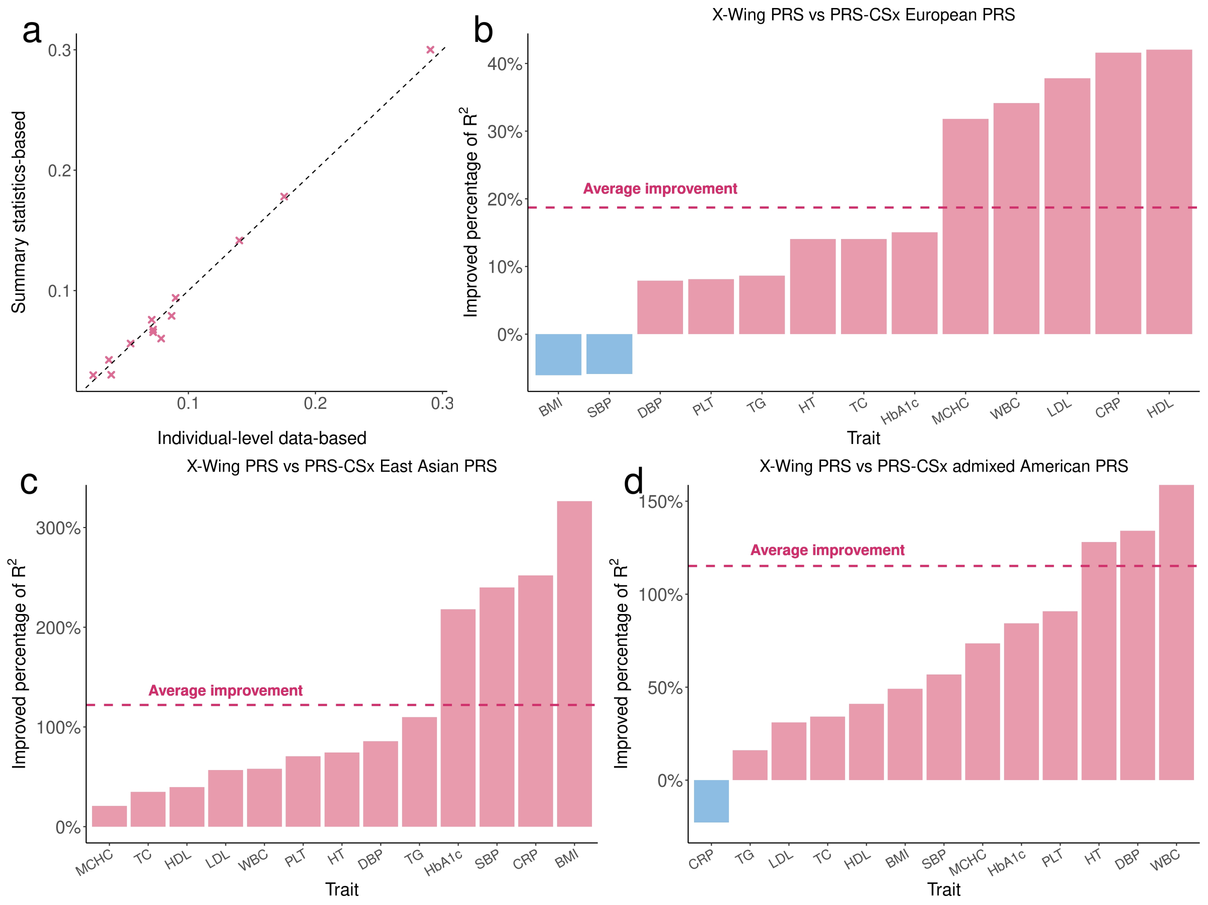

Supplementary Figure 20. Performance of X-Wing in combining population-specific PRS using GWAS summary statistics for 13 traits in admixed Americans. Panel **a** compares the R^2 for linearly combined PRS with mixing weights obtained using GWAS summary statistics and individual-level data. The X-axis represents the R^2 using weights estimated from individual-level data, while the Y-axis shows the R^2 using summary-statistics-based weights. The dashed line represents diagonal line of y=x. Panels **b, c**, and **d** show the percentage increase in R^2 of X-Wing PRS over PRS-CSx using only GWAS summary statistics, where the PRS-CSx PRS is calculated based on posterior mean effects of European, East Asian, and admixed American population, respectively. The dashed line represents the average increase.

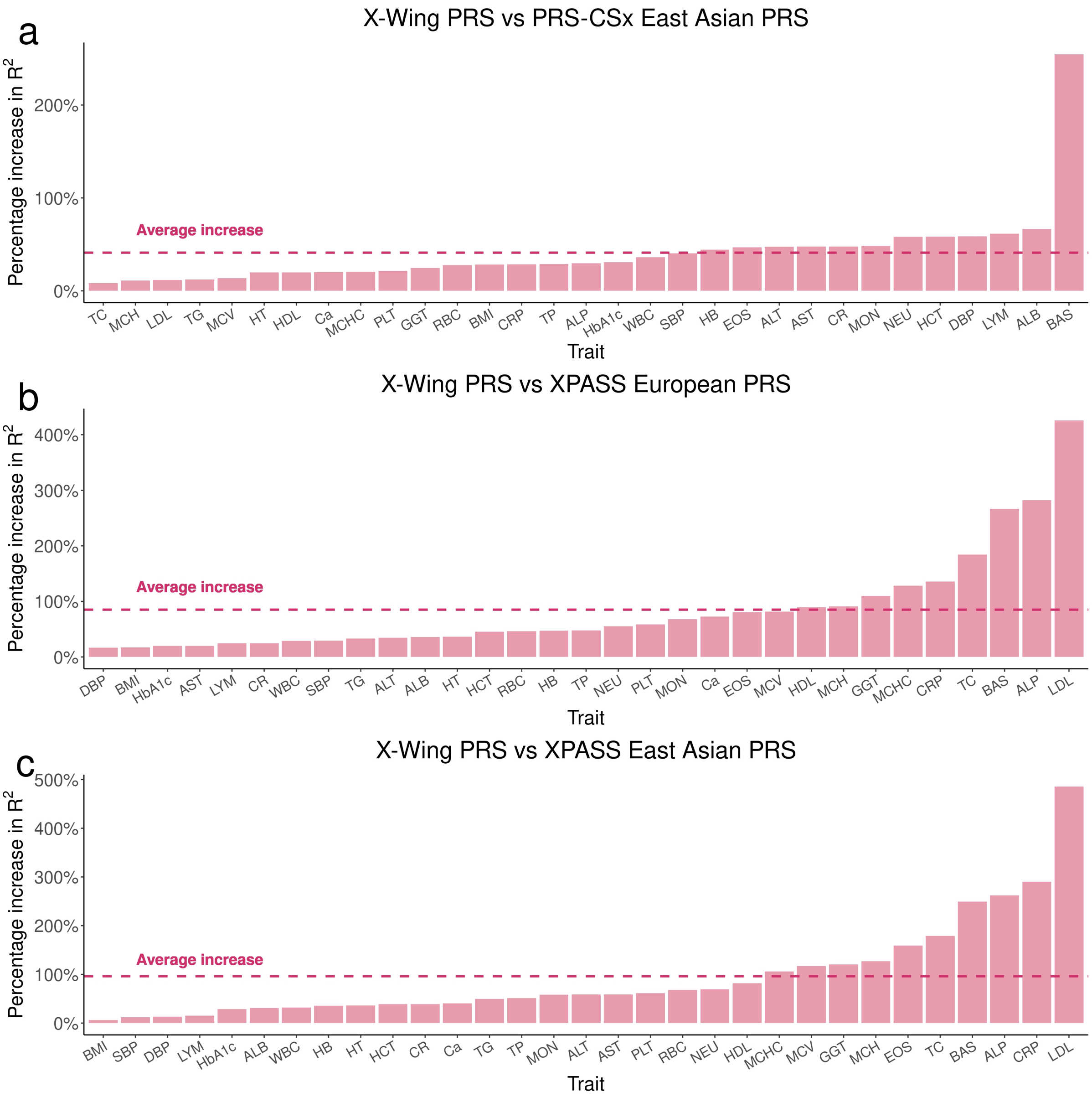

Supplementary Figure 21. Comparison of performance of summary statistics-based linearly combined X-Wing PRS with other methods for 31 traits in East Asians. It shows the percentage increase in R^2 of X-Wing PRS over PRS using **(a)** PRS-CSx East Asian ($P_{wilcoxon} = 1.8e - 9$) **(b)** XPASS European ($P_{wilcoxon} = 9.3e - 10$) **(c)** XPASS East Asian ($P_{wilcoxon} = 9.3e - 10$) posterior mean effects**.** The P-value is calculated using two-sided Wilcoxon signed-rank test.

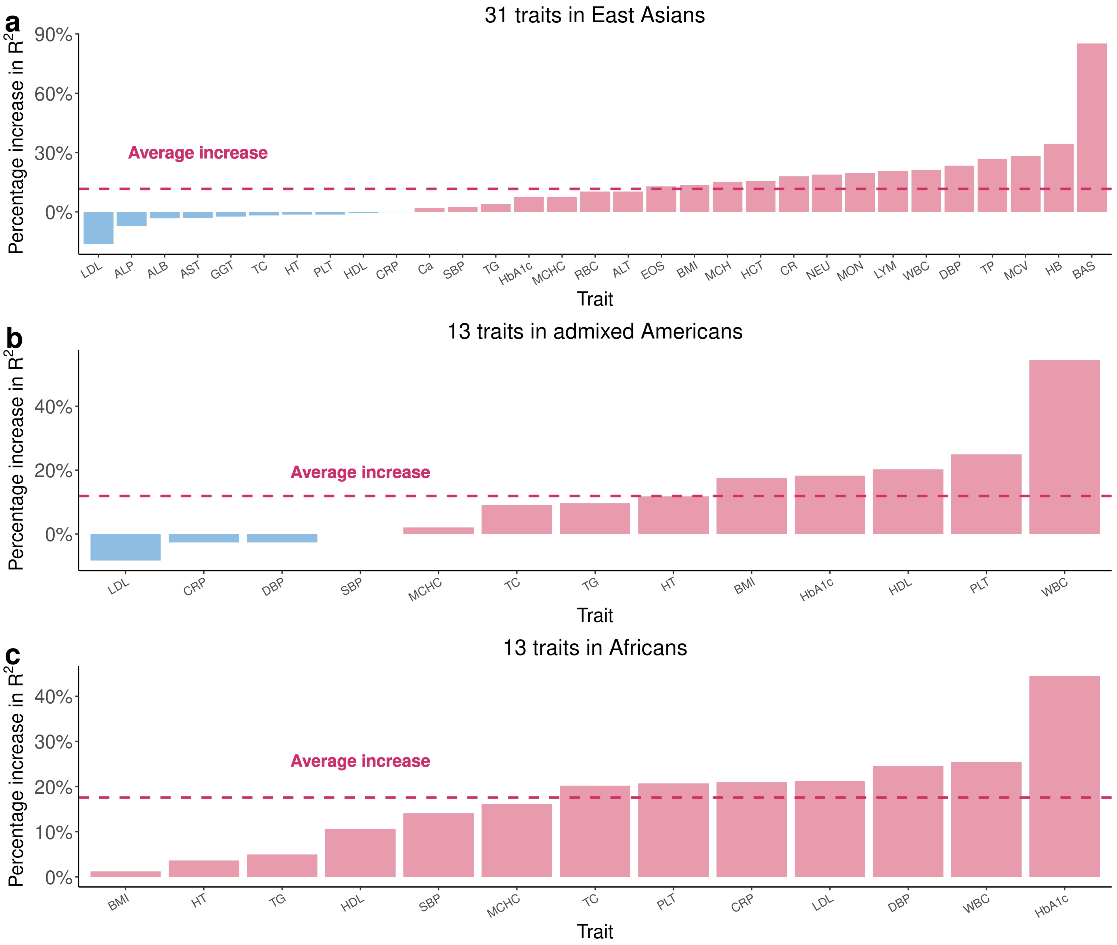

Supplementary Figure 22. Comparison of the prediction accuracy between X-Wing and PRS-CSx "-meta" PRS. a) 31 traits in East Asians **b)** 13 traits in admixed Americans **c)** 13 traits in Africans. X-Wing PRS is based on summary statistics-based linear combined PRS. The dashed line represents the average increase.

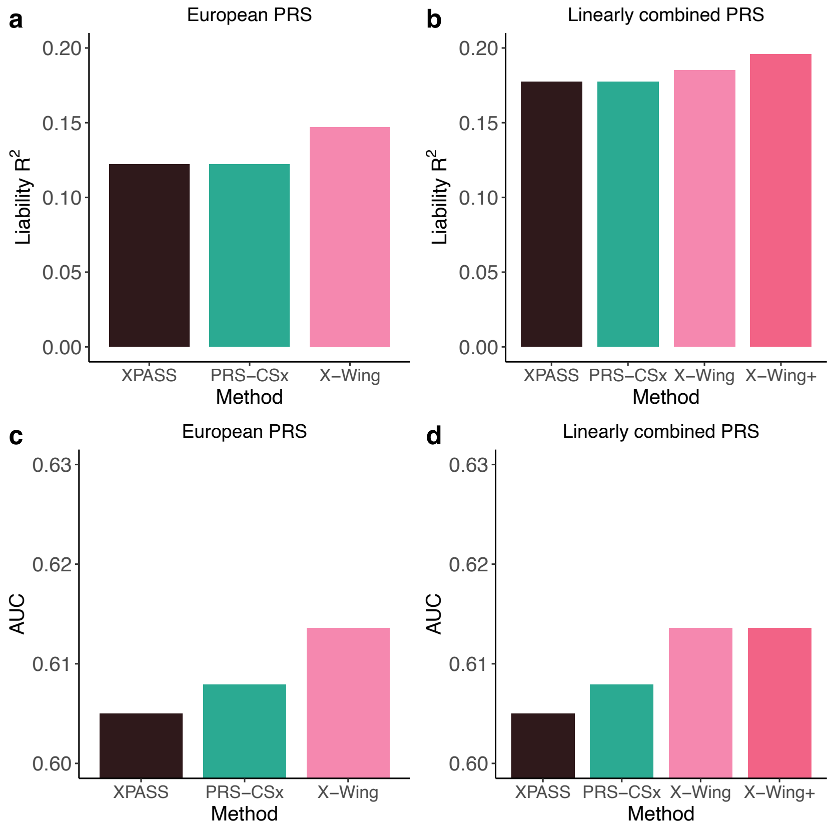

Supplementary Figure 23. Comparison of the prediction accuracy for type-2 diabetes in East Asians between (a) Liability R^2 of European PRS **(b)** Liability R^2 of linearly combined PRS **(c)** Area under the ROC Curve (AUC) of European PRS **(d)** AUC of linearly combined PRS.

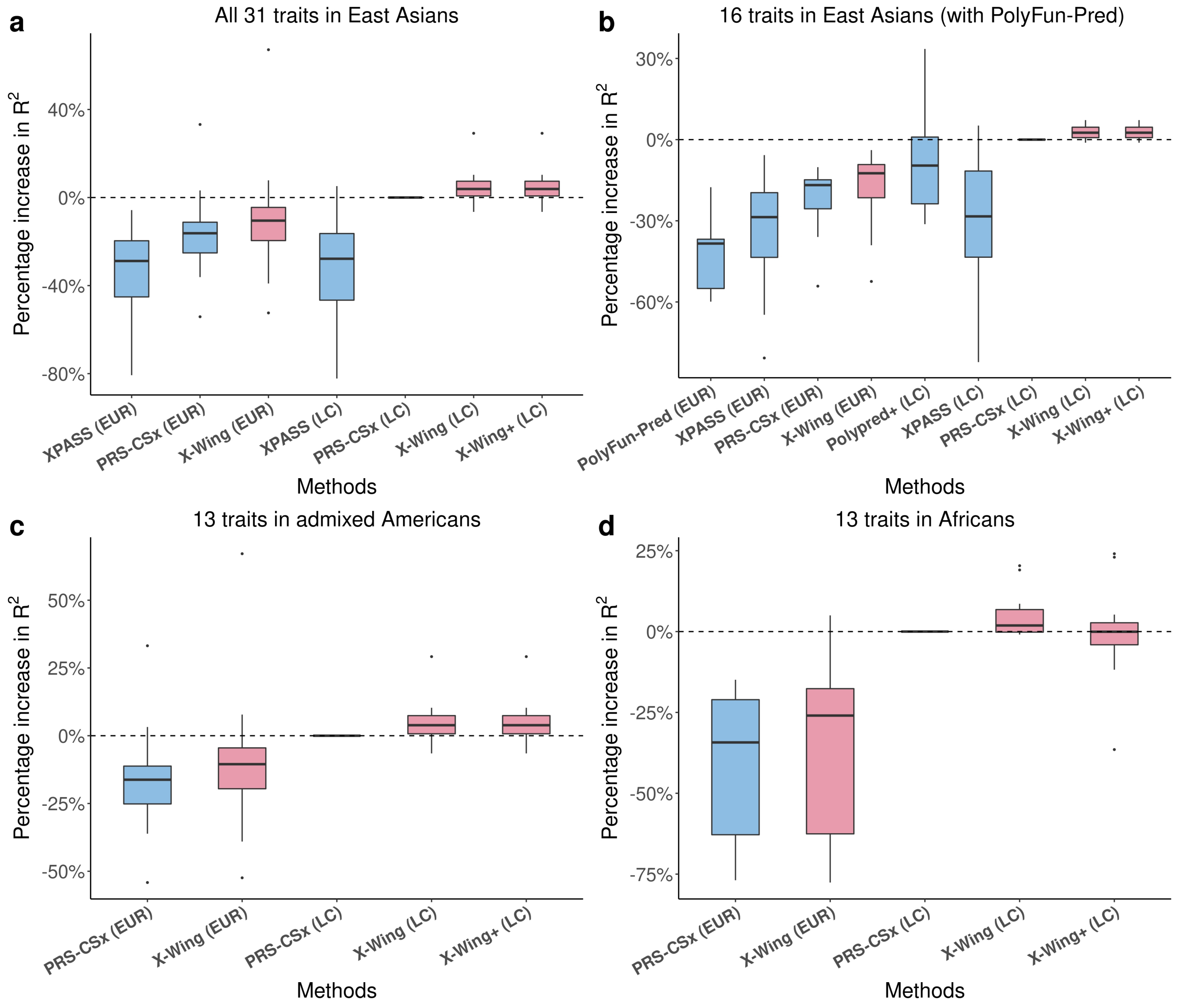

Supplementary Figure 24. Benchmark different methods' performance in different test sample. The test sample are **(a)** 31 traits in East Asians. **(b)** 16 traits in East Asians with PolyFun-Pred PRS coefficient available. **(c)** 13 traits in admixed Americans. **(d)** 13 traits in Africans. UKB and BBJ GWAS summary statistics are used as training data when the test sample are East Asians; UKB, BBJ and PAGE summary statistics are used as training data when the test sample are admixed Americans or Africans. "(EUR)" represents the PRS based on European posterior mean effects and "(LC)" represents the linearly combined PRS. "X-Wing (LC)" and "X-Wing+ (LC)" represents the X-Wing linearly combined PRS using mixing weights estimated from individual-level data and summary statistics-based repeated learning, respectively. "Polypred+ (LC)" linearly combines the effect sizes of BOLT-LMM-UKB, PolyFun-Pred, and BOLT-LMM-BBJ. In these boxplots, the center line, box limits and whiskers denote the median, upper and lower quartiles, and 1.5 × interquartile range, respectively.

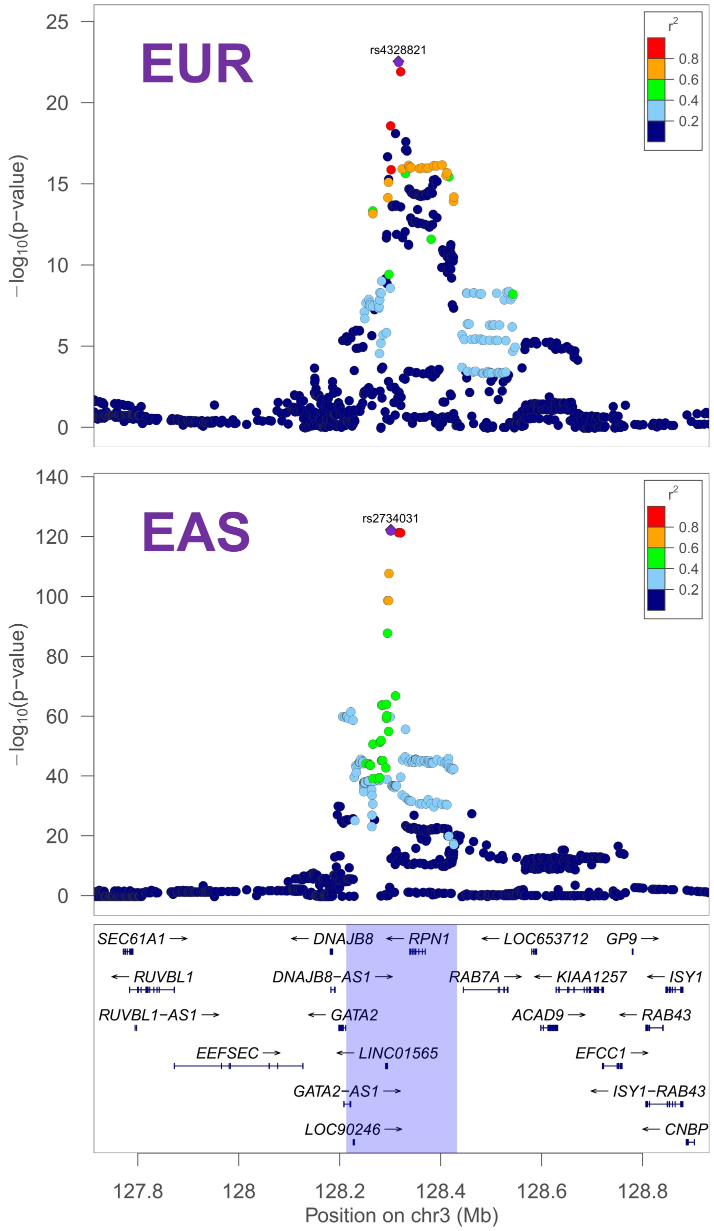

Supplementary Figure 25. Locuszoom plot of a locus on chromosome 3 between Europeans and East Asians for basophil count. The significant region is highlighted in blue. SNPs in this region achieve genome-wide significance in both populations (European lead SNP rs4328821, p = 2.82e-23; East Asian lead SNP rs2734031, p = 5.10e-123). The statistical test to get the P-value is two-sided t-test in marginal GWAS.

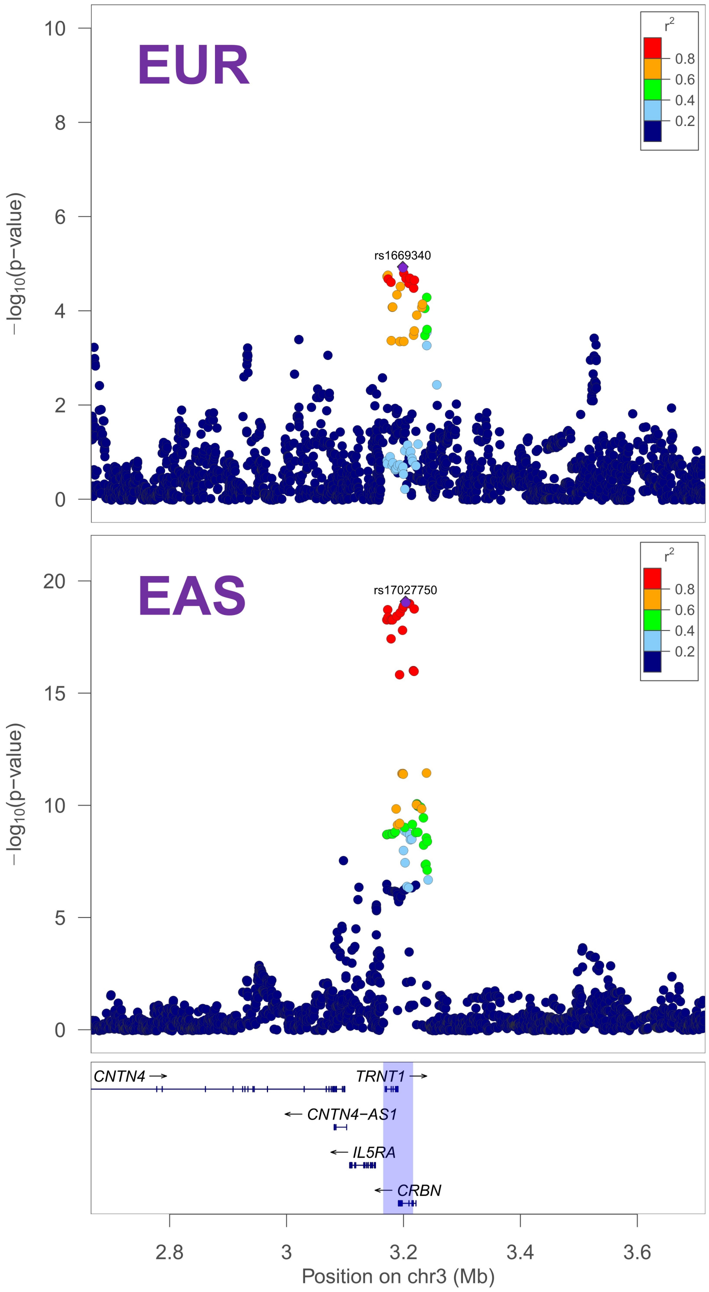

Supplementary Figure 26. Locuszoom plot of a locus on chromosome 3 between Europeans and East Asians for basophil count. The significant region is highlighted in blue. SNPs in this locus achieve genome-wide significance in East Asians (lead SNP rs17027750, p = 8.24e-20), but not in Europeans (lead SNP rs1669340, p = 1.16e-05). The statistical test to get the P-value is two-sided t-test in marginal GWAS.

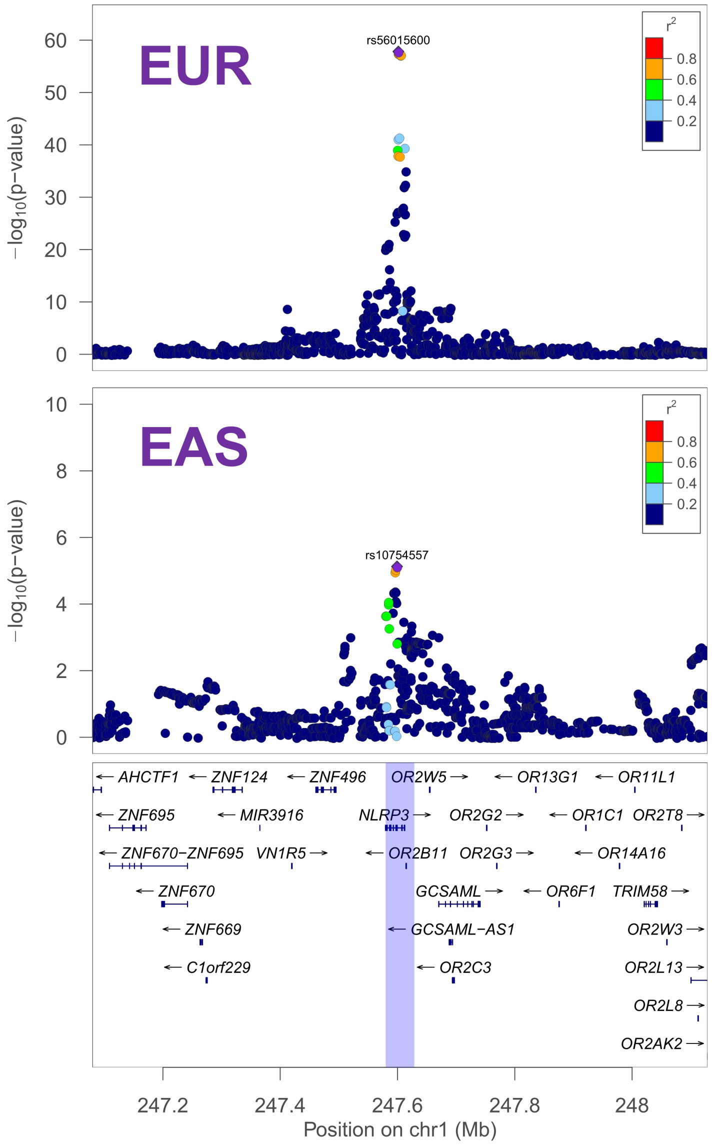

Supplementary Figure 27. Locuszoom plot of a locus on chromosome 1 between Europeans and East Asians for C-reactive protein. The significant region is highlighted in blue. SNPs in this locus achieve genome-wide significance in Europeans (lead SNP rs56015600, p = 1.48e-58), but not in East Asians (lead SNP rs10754557, p = 7.45e-6). The statistical test to get the P-value is two-sided t-test in marginal GWAS.

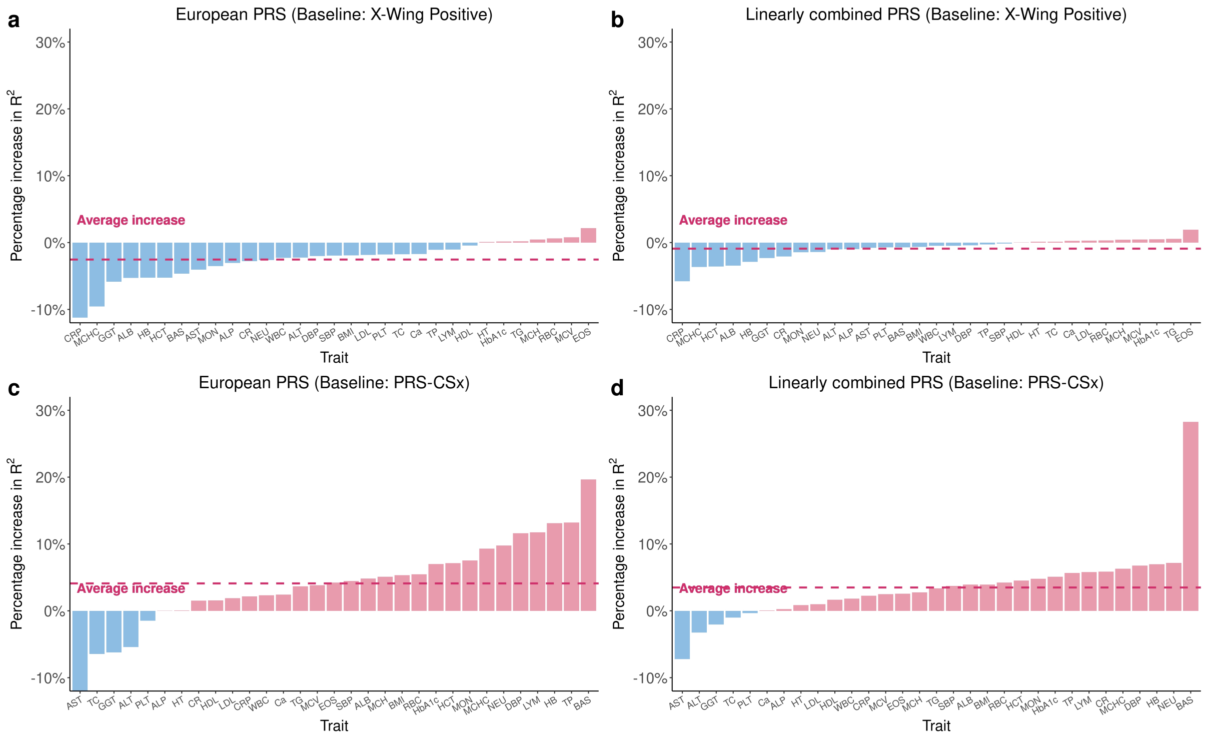

Supplementary Figure 28. Impact of using both positively and negatively correlated region as annotation on PRS prediction accuracy. (a) X-Wing + both positively and negatively correlated region vs X-Wing + only positively correlated region European PRS ($P_{wilcoxon} = 2.8e-5$). **(b)** X-Wing + both positively and negatively correlated region vs X-Wing + only positively correlated region linearly combined PRS ($P_{wilcoxon} = 0.02$). **(c)** X-Wing + both positively and negatively correlated vs PRS-CSx European PRS ($P_{wilcoxon} = 5.4e-4$). **(d)** X-Wing + both positively and negatively correlated vs PRS-CSx linearly combined PRS ($P_{wilcoxon} = 9.2e-6$). The dashed line represents the average decrease. The P-value is calculated using two-sided Wilcoxon signed-rank test.

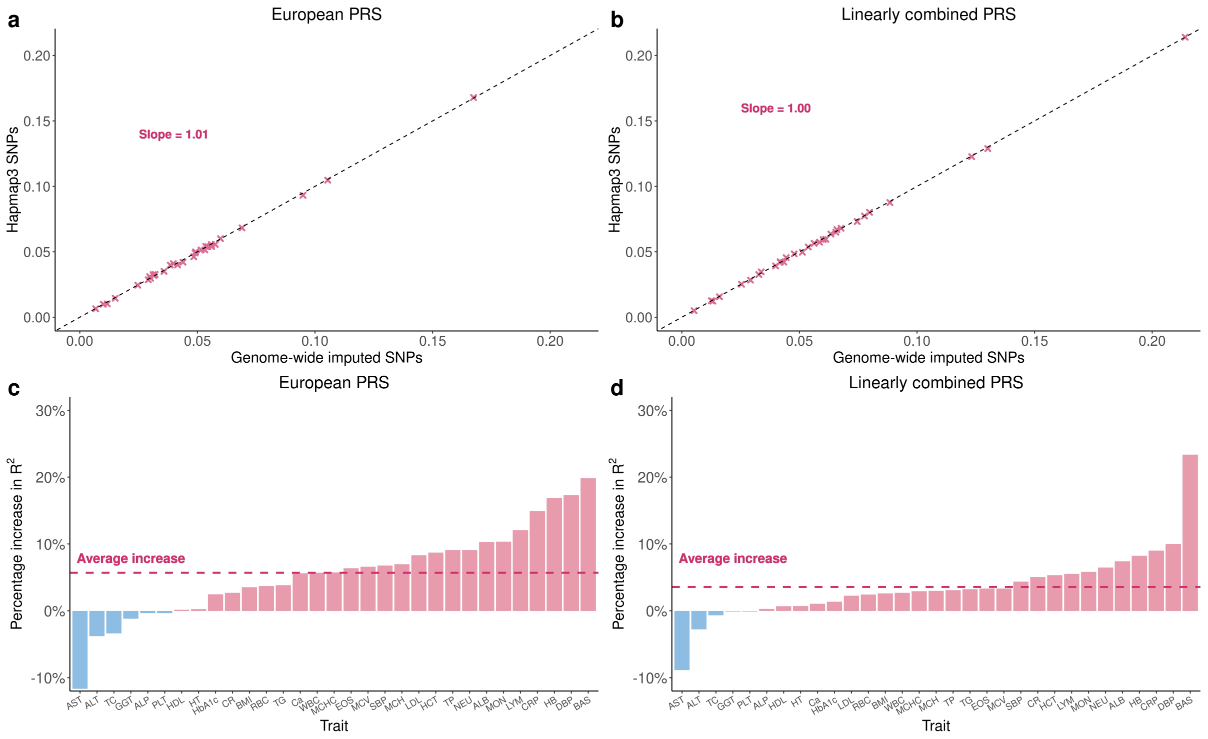

Supplementary Figure 29. Impact of using hapmap3 SNPs in identifying regions with local genetic correlation on PRS prediction accuracy for 31 traits in East Asians. (**a**) X-Wing (Hapmap3 SNPs annotation)vs X-Wing (Genome-wide imputed SNPs annotation) European PRS ($P_{wilcoxon} = 0.08$). (**b**) X-Wing (Hapmap3 SNPs annotation)+ vs X-Wing (Genome-wide imputed SNPs annotation) linearly combined PRS ($P_{wilcoxon} = 0.06$). (**c**) X-Wing (Hapmap3 SNPs annotation) vs PRS-CSx European PRS ($P_{wilcoxon} = 1.7e-5$). (**d**) X-Wing (Hapmap3 SNPs annotation) vs PRS-CSx linearly combined PRS ($P_{wilcoxon} = 3.5e-6$). All annotations are based on top 1000 positive regions. The dashed line represents the average decrease. The P-value is calculated using two-sided Wilcoxon signed-rank test.

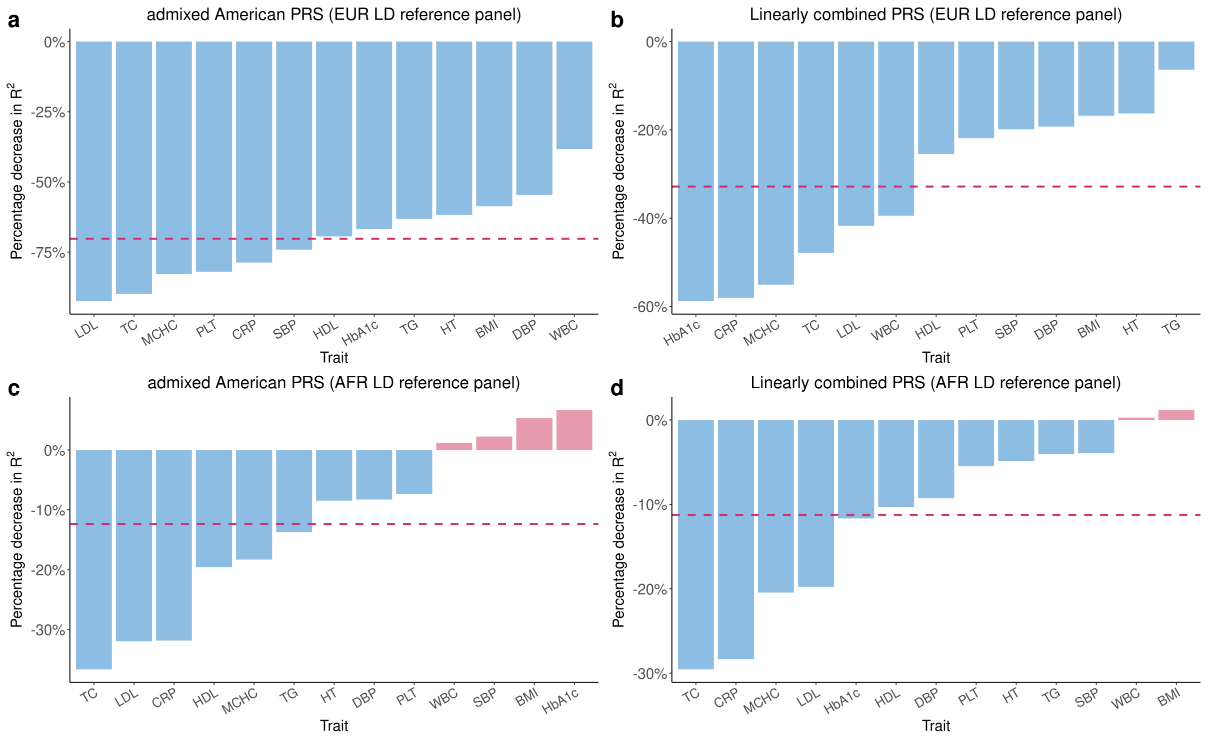

Supplementary Figure 30. Impact of mismatched LD reference panel on prediction accuracy for 13 traits in Africans. The baseline method is the X-Wing PRS using admixed American (AMR) LD reference panel. The methods in each figure are **(a)** X-Wing admixed American PRS using EUR LD reference panel (The P-value of two-sided Wilcoxon signed-rank test is $P_{wilcoxon} = 2.4e-4$). **(b)** X-Wing linearly combined PRS using EUR LD reference panel ($P_{wilcoxon} = 2.4e-4$). **(c)** X-Wing admixed American PRS using AFR LD reference panel ($P_{wilcoxon} = 0.017$). **(d)** X-Wing linearly combined PRS using AFR LD reference panel ($P_{wilcoxon} = 1.2e-3$). The dashed line represents the average decrease. The P-value is calculated using two-sided Wilcoxon signed-rank test.

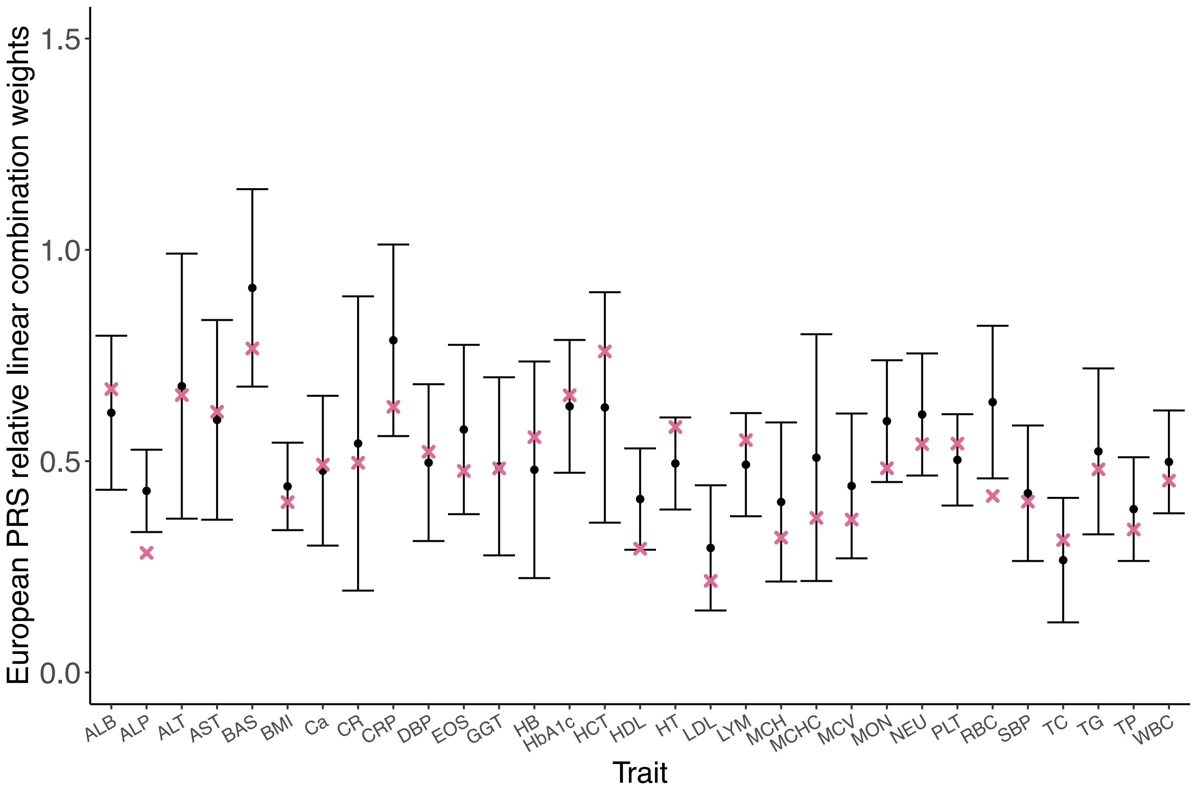

Supplementary Figure 31. Comparison of linear combination weights estimated from summary statistics and individual-level validation data for 31 traits in East Asians. The black point represents the mean of estimated weights across 100 replicates. The error bar represents the 95% confidence interval. The pink cross point is the weights estimated from summary statistics-repeated learning.

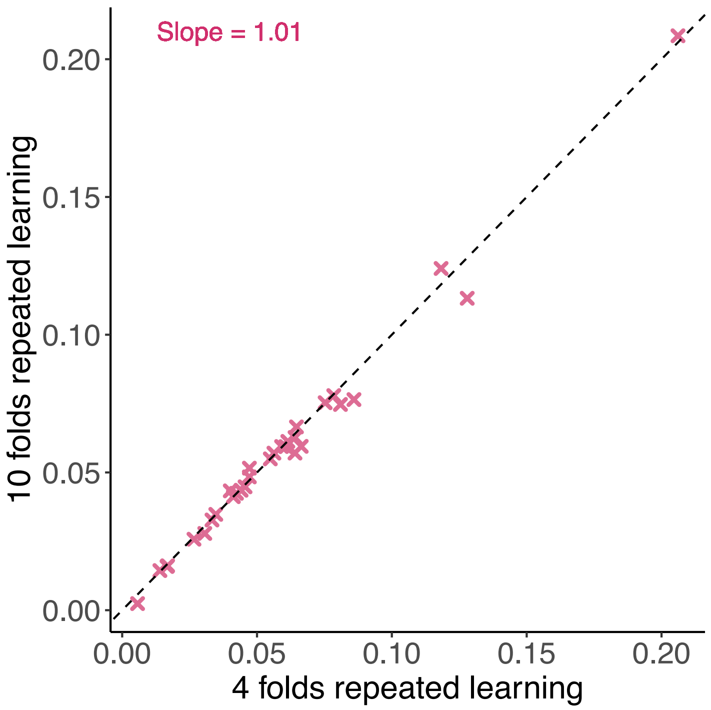

Supplementary Figure 32. Impact of number of repeated learning folds on prediction accuracy of X-Wing linearly combined PRS with mixing weights obtained using GWAS summary statistics for 31 traits in East Asians. X-axis represents 4 folds and Y-axis is 10 folds repeated learning. The P-value of two-sided Wilcoxon signed-rank test is $P_{wilcoxon} = 0.14$.

Supplementary Methods

Contents

1 Identification of genomic regions showing local genetic correlations between two ancestral populations 2

2 An annotation-dependent Bayesian horseshoe regression model for PRS 3

2.1 Incorporating local genetic correlation annotation in PRS 3

2.1.1 Model 3

2.1.2 Gibbs sampler 4

2.1.3 Example of annotation-dependent shrinkage based on local genetic correlation annotation 6

2.1.4 Model-tuning strategy 7

2.2 Incorporating multiple annotations 8

2.2.1 Model 8

2.2.2 Gibbs sampler 8

3 Combining multiple PRS with GWAS summary statistics 9

3.1 Sufficient statistics for least squares estimator of linear combination weights 9

3.2 Derivation for subsampling GWAS summary statistics from training and validation sets 10

3.3 Dealing with tuning parameters in the PRS model 12

3.4 Grid search to handle negative least squares estimates for mixing weights 13

3.5 Summary statistics-based ridge regression to combine multiple PRS 13

3.6 Transforming allele count scale SNP effects into standardized scale 14

3.7 GWAS summary statistics-based cross-validation 15

3.8 Regarding the marginal linear assumptions 15

4 Implementation of other methods 16

5 Simulation details 17

6 Computational cost 17

7 Supplementary References 19

1 Identification of genomic regions showing local genetic correlations between two ancestral populations

We have recently developed LOGODetect, which can precisely identify local, genetically-correlated regions between two traits in single population. Here we extend LOGODetect to detect genomic regions enriched for cross-population local genetic correlations.

Suppose the standardized traits $\boldsymbol{Y}_1$ and $\boldsymbol{Y}_2$ in two populations follow the linear models with random effects:

$$\boldsymbol{Y}_k = \boldsymbol{X}_k\boldsymbol{\beta}_k + \boldsymbol{\epsilon}_k, k = 1, 2, \tag{1}$$

where $\boldsymbol{X}_k$ is a $N_k \times M$ standardized genotype matrix; $\boldsymbol{\beta}_k$ is a M-dimensional vector of genetic effect sizes; $\boldsymbol{\epsilon}_k$ are non-genetic effects. We assume cross-population genetic covariance is localized in small regions $R_1, \dots, R_r$, i.e. the joint genetic effect sizes follow the multivariate normal distribution:

$$\begin{bmatrix} \boldsymbol{\beta}_1 \\ \boldsymbol{\beta}_2 \end{bmatrix} \sim \mathcal{N}\left(\begin{bmatrix} \mathbf{0} \\ \mathbf{0} \end{bmatrix}, \begin{bmatrix} \frac{h_1^2}{M}\boldsymbol{I} & \frac{\rho_g}{K}\tilde{\boldsymbol{I}} \\ \frac{\rho_g}{K}\tilde{\boldsymbol{I}} & \frac{h_2^2}{M}\boldsymbol{I} \end{bmatrix}\right), \tag{2}$$

where h_1^2 and h_2^2 denote heritability in two populations; ρ_g is the cross-population genetic covariance; $\tilde{\boldsymbol{I}}$ is a diagonal matrix where $\tilde{\boldsymbol{I}}[i,i] = 1$ if and only if $i \in \cup_{j=1}^r R_j$; $K = \sum_{j=1}^r |R_j|$, which is the number of SNPs with correlated genetic effect. We assume non-genetic effects $\boldsymbol{\epsilon}_k$ are independent across two populations.

Our goal is to use scan statistic to identify small regions enriched for local genetic correlation between two populations. Therefore, we design the numerator in the scan statistic as $\sum_{i\in R} z_{1i}z_{2i}$, the inner product of z-scores in a region across two populations, which quantifies the concordant association pattern of SNP effect sizes. To normalize the effects of LD in two populations, we use $\mathrm{Sd}\left[\sum_{i\in R} z_{1i}z_{2i}\right]$ under the null hypothesis as the denominator of scan statistic. Under the null hypothesis that cross-population genetic correlation is zero, the joint z-scores for two populations follow the multivariate normal distribution as

$$\begin{bmatrix} \boldsymbol{z}_1 \\ \boldsymbol{z}_2 \end{bmatrix} \sim \mathcal{N}\left(\begin{bmatrix} 0 \\ 0 \end{bmatrix}, \begin{bmatrix} \frac{\frac{h_1^2}{M}\boldsymbol{X}_1^T\boldsymbol{X}_1\boldsymbol{X}_1^T\boldsymbol{X}_1 + \left(1-h_1^2\right)\boldsymbol{X}_1^T\boldsymbol{X}_1}{n_1} & \mathbf{0} \\ \mathbf{0} & \frac{\frac{h_2^2}{M}\boldsymbol{X}_2^T\boldsymbol{X}_2\boldsymbol{X}_2^T\boldsymbol{X}_2 + \left(1-h_2^2\right)\boldsymbol{X}_2^T\boldsymbol{X}_2}{n_2} \end{bmatrix}\right). \tag{3}$$

Since individual genotype data is hardly accessible due to privacy issues in practice, we use LD matrices (denoted by $\boldsymbol{V}_k$) estimated from reference panels (e.g. the European and East Asian individuals from 1000 Genome Project) to approximate the sample LD matrices $\frac{\boldsymbol{X}_k^T\boldsymbol{X}_k}{N_k}$. Further, $\frac{\boldsymbol{X}_k^T\boldsymbol{X}_k\boldsymbol{X}_k^T\boldsymbol{X}_k}{N_k^2}$ can be unbiasedly estimated as $\widetilde{\boldsymbol{V}_k^2} = \frac{N_k^{(ref)}-1}{N_k^{(ref)}-2}\boldsymbol{V}_k^2 - \frac{M}{N_k^{(ref)}-2}\boldsymbol{V}_k$, where $N_k^{(ref)}$ is the sample size of the reference panel for population k. Using this approximation, we have

$$\begin{bmatrix} \boldsymbol{z}_1 \\ \boldsymbol{z}_2 \end{bmatrix} \sim \mathcal{N}\left(\begin{bmatrix} 0 \\ 0 \end{bmatrix}, \begin{bmatrix} \frac{N_1h_1^2}{M}\widetilde{\boldsymbol{V}_1^2} + \left(1-h_1^2\right)\boldsymbol{V}_1 & 0 \\ 0 & \frac{N_2h_2^2}{M}\widetilde{\boldsymbol{V}_2^2} + \left(1-h_2^2\right)\boldsymbol{V}_2 \end{bmatrix}\right). \tag{4}$$

We use XPASS[1] to estimate heritability for two populations h_1^2 and h_2^2. Let $\boldsymbol{\Sigma}_k = \frac{N_kh_k^2}{M}\widetilde{\boldsymbol{V}_k^2} + \left(1-h_k^2\right)\boldsymbol{V}_k$. Denote $\boldsymbol{z}_{kR}$ as the sub-vector of $\boldsymbol{z}_k$ indexed by R, and $\boldsymbol{\Sigma}_{k,RR}$ as the sub-matrix of $\boldsymbol{\Sigma}_k$ whose rows and columns are both

indexed by R. Then for a given region R, the joint z-scores in R follows the multivariate normal distribution

$$\begin{bmatrix} \boldsymbol{z}_{1R} \\ \boldsymbol{z}_{2R} \end{bmatrix} \sim \mathcal{N}\left(\begin{bmatrix} \mathbf{0} \\ \mathbf{0} \end{bmatrix}, \begin{bmatrix} \boldsymbol{\Sigma}_{1,RR} & \mathbf{0} \\ \mathbf{0} & \boldsymbol{\Sigma}_{2,RR} \end{bmatrix}\right). \tag{5}$$

Following bilinear form theory, we can show that $\mathrm{Var}\left[\sum_{i\in R} z_{1i} z_{2i}\right] = \mathrm{Tr}\left[\boldsymbol{\Sigma}_{1,RR}\boldsymbol{\Sigma}_{2,RR}\right]$, where $\mathrm{Tr}[\boldsymbol{A}]$ denotes the trace of matrix $\boldsymbol{A}$. However, computing value of $\mathrm{Tr}\left[\boldsymbol{\Sigma}_{1,RR}\boldsymbol{\Sigma}_{2,RR}\right]$ for all possible regions R is computationally expensive. Therefore, we use $\sum_{i\in R} \boldsymbol{\Sigma}_{1,ii} * \boldsymbol{\Sigma}_{2,ii}$ to replace $\mathrm{Tr}\left[\boldsymbol{\Sigma}_{1,RR}\boldsymbol{\Sigma}_{2,RR}\right]$. We add tuning parameter θ to the power term of $\sum_{i\in R} \boldsymbol{\Sigma}_{1,ii} * \boldsymbol{\Sigma}_{2,ii}$ to accommodate the approximation bias and control the penalty strength of LD effects.

We select the best tuning parameter θ from a candidate set {0.5, 0.55, 0.6, 0.65, 0.7, 0.75} such that the identified regions for that given θ achieve the highest proportion of genetic covariance. The cross-population genetic covariance for aggregated regions can be estimated using XPASS[1].

We compute the cross-population LD matrix by taking the maximum of population-specific LD matrix in an element-wise fashion, as previously suggested[2]. We use ldetect[3] to divide the genome into 185 LD blocks (average size of 15MB) that are approximately independent in both populations based on the cross-population LD matrix. We first apply LOGODetect to identify regions with family-wise error rate cutoff of 0.05 in each LD block separately. Then we collect all the candidate regions identified across different LD blocks and control FDR level of 0.05 using the Benjamini-Hochberg procedure.

2 An annotation-dependent Bayesian horseshoe regression model for PRS

2.1 Incorporating local genetic correlation annotation in PRS

2.1.1 Model

Consider an additive genetic model:

$$\boldsymbol{Y}_k = \boldsymbol{X}_k \boldsymbol{\beta}_k + \boldsymbol{\epsilon}_k, \quad \boldsymbol{\epsilon}_k \sim \mathcal{MVN}\left(\mathbf{0}, \sigma_k^2 \boldsymbol{I}_k\right), \quad p\left(\sigma_k^2\right) \propto \sigma_k^{-2}, \quad k = 1, 2, \cdots, K, \tag{6}$$

where $\boldsymbol{\beta}_k$ is a M-dimensional vector of SNP effect sizes in population $k, \boldsymbol{\epsilon}_k$ is a vector of error terms with variance σ_k^2, to which we assign a non-informative Jeffreys prior. $\mathcal{MVN}$ denotes multivariate normal distribution, and $\boldsymbol{I}_k$ is an identity matrix.

Consider an annotation with A category, we assign an annotation-dependent horseshoe prior to β_{jk}:

$$\beta_{jk} \sim \mathcal{N}\left(0, \frac{\sigma_k^2}{N_k} \phi \psi_j \lambda_{f(j),k}\right), \ j = 1, \ldots, M, \ k = 1, \ldots, K. \tag{7}$$

Here, β_{jk} denotes the effect of SNP j in population k, ϕ is the global shrinkage parameter shared across all M SNPs, ψ_j represents the local shrinkage parameters for SNP j, $\lambda_{f(j),k}$ denotes the annotation-dependent

shrinkage parameter for SNP j in population $k, f : j \to a \in \{1, \dots A\}$ is a function that maps the j-th SNP to its corresponding category a in the annotation.

To perform the full Bayesian model fitting, we assign the half-Cauchy priors to the global, local, and annotation-dependent shrinkage parameters as follows:

$$\begin{aligned} &\phi^{\frac{1}{2}} \sim C^{+}(0,1) \\ &\psi_j^{\frac{1}{2}} \sim C^{+}(0,1) \quad j = 1, \dots, M \\ &\lambda_{a,k}^{\frac{1}{2}} \sim C^{+}(0,1) \quad a = 1, \dots, A, k = 1, \dots, K. \end{aligned} \tag{8}$$

Using the half-Cauchy decomposition, we have

$$\begin{aligned} \phi \mid v &\sim \mathcal{IG}\left(\frac{1}{2}, \frac{1}{v}\right), v \sim \mathcal{IG}\left(\frac{1}{2}, 1\right) \\ \psi_j \mid c_j &\sim \mathcal{IG}\left(\frac{1}{2}, \frac{1}{c_j}\right), c_j \sim \mathcal{IG}\left(\frac{1}{2}, 1\right) \quad j = 1, \dots, M \\ \lambda_{a,k} \mid t_{a,k} &\sim \mathcal{IG}\left(\frac{1}{2}, \frac{1}{t_{a,k}}\right), t_{a,k} \sim \mathcal{IG}\left(\frac{1}{2}, 1\right) \quad a = 1, \dots, A, \end{aligned} \tag{9}$$

where $\mathcal{IG}$ denotes the inverse-gamma distribution.

2.1.2 Gibbs sampler

Next, we derive the full conditional distribution of all parameters in the above model.

For notation purpose, we rewrite the prior in matrix form:

$$\boldsymbol{\beta}_k \sim \mathcal{N}\left(0, \frac{\sigma_k^2}{N_k} \phi \boldsymbol{\Psi} \boldsymbol{\Lambda}_k\right), \ j = 1, \dots, M, \ k = 1, \dots, K, \tag{10}$$

where $\boldsymbol{\Psi} = \operatorname{diag}(\psi_1, \dots, \psi_M), \boldsymbol{\Lambda}_k = \operatorname{diag}\left(\lambda_{f(1),k}, \dots, \lambda_{f(M),k}\right)$.

The Gibbs sampler involves the following steps in each Markov Chain Monte Carlo (MCMC) iteration:

- update $\boldsymbol{\beta}_k : \boldsymbol{\beta}_k \mid \cdot \sim \mathcal{MVN}(\mu_k, \boldsymbol{\Sigma}_k), \quad \mu_k = \frac{N_k}{\sigma_k^2} \boldsymbol{\Sigma}_k \hat{\boldsymbol{\beta}}_k, \quad \boldsymbol{\Sigma}_k = \frac{\sigma_k^2}{N_k} \left(\boldsymbol{D}_k + (\phi \boldsymbol{\Psi} \boldsymbol{\Lambda}_k)^{-1}\right)^{-1},$

  where D_k is the LD-matrix for population k, $\hat{\boldsymbol{\beta}}_k$ is the marginal least squares estimates obtained from GWAS summary statistics. To avoid the numerical issue caused by colinearity between SNPs, we restrict $(\phi \boldsymbol{\Psi} \boldsymbol{\Lambda}_k)^{-1} \geq 1$.

- update $\sigma_k^2 : \sigma_k^2 \mid \cdot \sim \mathcal{IG}\left(\frac{N_k + M_k}{2}, \frac{N_k}{2}\left[1 - 2\boldsymbol{\beta}_k^T \hat{\boldsymbol{\beta}}_k + \boldsymbol{\beta}_k^T \left(\boldsymbol{D}_k + \phi \boldsymbol{\Psi} \boldsymbol{\Lambda}_k\right) \boldsymbol{\beta}_k\right]\right),$

  where M_k is number of SNPs in population k.

- update $\phi : \phi \mid \cdot \sim \mathcal{IG}\left(\frac{\sum_{k=1}^K M_k + 2}{2}, \sum_{k=1}^K \frac{\boldsymbol{\beta}_k^T (\boldsymbol{\Psi} \boldsymbol{\Lambda}_k)^{-1} \boldsymbol{\beta}_k N_k}{2\sigma_k^2} + \frac{1}{\nu}\right)$

- update $\nu : \nu \mid \cdot \sim \mathcal{IG}\left(1, \frac{1}{\phi} + 1\right)$

- update $\psi_j : \psi_j \mid \cdot \sim \mathcal{IG}\left(\frac{k_j + 1}{2}, \sum_{k=1}^K \frac{\beta_{jk}^2 N_k}{2\sigma_k^2 \phi \lambda_{f(j),k}} + \frac{1}{c_j}\right)$

  where $k_j = 1$ if SNP j exists only in one population and r if it exists in r populations included.

- update $c_j : c_j \mid \cdot \sim \mathcal{IG}\left(1, \frac{1}{\psi_j} + 1\right)$
- update $\lambda_{a,k} : \lambda_{a,k} \mid \cdot \sim \mathcal{IG}\left(\frac{s_q+1}{2}, \frac{1}{2\phi} \sum_{j \in l(a,k)} \frac{\beta_{jk}^2 N_k}{\psi_j \sigma_k^2} + \frac{1}{t_{a,k}}\right)$

  where s_a is the number of predictors in category a, $l(a,k) = \{j \in \{1, \ldots, M\} : \lambda_{f(j),k} = \lambda_{a,k}\}$ is the set of SNP predictors that belongs to category a.
- update $t_{a,k} \mid \cdot \sim \mathcal{IG}\left(1, \frac{1}{\lambda_{a,k}} + 1\right)$

2.1.3 Example of annotation-dependent shrinkage based on local genetic correlation annotation

Here, we provide an example of the annotation-dependent shrinkage $\lambda_{f(j),k}$ based on local genetic correlation annotation. WLOG, we assume that we have $K = 3$ populations in total and population 1 is the target population.

Given the local genetic correlation annotation Ω_2 and Ω_3, the $\lambda_{f(j),k}$ in the Full Bayesian model fitting process is specified as:

Annotation-dependent shrinkage $\lambda_{f(j),k}$	**Population 1 (target)**	Population 2	Population 3
Posterior effects for population 1	1 for all j	1 for all j	1 for all j
Posterior effects for population 2	$\lambda_{1,1}$ for all j	$\lambda_{1,2}$ if SNP j is not annotated by Ω_2<br>$\lambda_{2,2}$ if SNP j is annotated by Ω_2	$\lambda_{1,3}$ for all j
Posterior effects for population 3	$\lambda_{1,1}$ for all j	$\lambda_{1,2}$ for all j	$\lambda_{1,3}$ if SNP j is not annotated by Ω_3<br>$\lambda_{2,3}$ if SNP j is annotated by Ω_3

Supplementary Table 1: Example of annotation-dependent shrinkage based on local genetic correlation annotation

Here, k-th row represents the specification of the annotation-dependent shrinkage parameter $\lambda_{f(j),k'}$ for $k^{'} = 1, 2, 3$ when obtaining the posterior effects for k-th population.

2.1.4 Model-tuning strategy

Instead of assigning a prior for ϕ, we select the global shrinkage parameter ϕ from a grid of value $\{10^{-6}, 10^{-4}, 10^{-2}, 1\}$ with respect to the largest R^2 in the validation set. The detailed algorithm is listed below:

Algorithm 1: Model-tuning X-Wing

Input: GWAS summary statistics and population-matached LD reference panel from population 1 to K, target sample genotype.

Output: X-Wing PRS.

1 We perform local genetic correlation analysis between population 1 and population k $(k = 2, \dots K)$ to identify top s regions with positive local genetic correlation. We denote the set of regions as Ω_k.

2 For each $\phi \in \{10^{-6}, 10^{-4}, 10^{-2}, 1\}$, we fit our PRS model with annotation-dependent shrinkage specified below: when estimating the posterior SNP effects for the non-target population k that $k \neq 1$, we used $\lambda_{f(j),k} = \lambda_{1,k}$ if SNP j is not annotated by Ω_k, $\lambda_{f(j),k} = \lambda_{2,k}$ if SNP j is annotated by Ω_k, and $\lambda_{f(j),k'} = 1$ for $k^{'} = 1, 2, \dots, k-1, k+1, \dots, K$. When estimating the posterior SNP effects for target population, we used $\lambda_{f(j),k} = 1$ for all $j = 1, 2, \dots M, k = 1, \dots K$.

3 For each $\phi \in \{10^{-6}, 10^{-4}, 10^{-2}, 1\}$, based on the posterior mean effects of population k obtained in step2, we calculate population-specific score $\boldsymbol{PRS}_{k,\phi}$. A common practice to combine these population-specific scores is to fit a regression model using the same phenotype $\boldsymbol{Y}_1^{(v)}$ and K population-specific PRS in an independent validation dataset from the target population:

$$\boldsymbol{Y}_1^{(v)} \sim w_{1,\phi}\boldsymbol{PRS}_{1,\phi}^{(v)} + w_{2,\phi}\boldsymbol{PRS}_{2,\phi}^{(v)} + \cdots + w_{K,\phi}\boldsymbol{PRS}_{K,\phi}^{(v)}, \tag{11}$$

Instead of fitting a regression in independent samples, we employ a novel strategy to obtain the least squares estimates of regression weights (i.e. $\widehat{w}_{1,\hat{\phi}}, \dots \widehat{w}_{K,\hat{\phi}}$) using GWAS summary statistics. We introduce this approach in the section 3.3.

4 The final X-Wing PRS is then calculated as:

$$\boldsymbol{PRS}_{LC} = \sum_{k=1}^{K} \widehat{w}_{k,\hat{\phi}}\boldsymbol{PRS}_{k,\hat{\phi}} \tag{12}$$

2.2 Incorporating multiple annotations

2.2.1 Model

We generalized our model to incorporate T annotations with the annotation-dependent shrinkage prior to β_{jk}:

$$\beta_{jk} \sim \mathcal{N}\left(0, \frac{\sigma_k^2}{N_k}\phi_k\psi_j\prod_{t=1}^{T}\lambda_{f(j,t),k}\right),\ j=1,\ldots,M,\ k=1,\ldots,K. \tag{13}$$

Here, β_{jk} denotes the effect of SNP j in population k, ϕ_k is the global shrinkage parameter shared across all SNPs for population k, ψ_j represents the local shrinkage parameters for SNP j and are shared across population, $\lambda_{f(j,t),k}$ is the annotation-dependent shrinkage parameters for SNP j in population k for t-th annoration, $f:(j,t)\to a_t \in \{1,\ldots,\ldots A_t\}$ is a function that maps the j-th SNP to its corresponding category a_t in the t-th annotation.

To perform the full Bayesian model fitting, we assign the half-Cauchy priors to the global, local, and annotation-dependent shrinkage parameters as follows:

$$\begin{aligned}
&\phi_k^{\frac{1}{2}} \sim C^+(0,1) \quad k=1,\ldots,K\\
&\psi_j^{\frac{1}{2}} \sim C^+(0,1) \quad j=1,\ldots,M\\
&\lambda_{a_t,k}^{\frac{1}{2}} \sim C^+(0,1) \quad a_t=1,\ldots,A_t, t=1,\ldots,T, k=1,\ldots,K
\end{aligned} \tag{14}$$

Using the half-Cauchy decomposition, we have

$$\begin{aligned}
\phi_k \mid v &\sim \mathcal{IG}\left(\frac{1}{2},\frac{1}{v_k}\right), v_k \sim \mathcal{IG}\left(\frac{1}{2},1\right)\\
\psi_j \mid c_j &\sim \mathcal{IG}\left(\frac{1}{2},\frac{1}{c_j}\right), c_j \sim \mathcal{IG}\left(\frac{1}{2},1\right) \quad j=1,\ldots,M\\
\lambda_{a_t,k} \mid t_{a_t,k} &\sim \mathcal{IG}\left(\frac{1}{2},\frac{1}{t_{a_t,k}}\right), t_{a_t,k} \sim \mathcal{IG}\left(\frac{1}{2},1\right) \quad a_t=1,\ldots,A_t, k=1,\ldots,K,
\end{aligned} \tag{15}$$

where $\mathcal{IG}$ denotes the inverse-gamma distribution.

2.2.2 Gibbs sampler

Next, we derive the full conditional distribution of all parameters in the above model.

For notation purpose, we rewrite the prior in matrix form:

$$\boldsymbol{\beta}_k \sim \mathcal{N}\left(0, \frac{\sigma_k^2}{N_k}\phi_k\boldsymbol{\Psi}\boldsymbol{\Lambda}_{k1}\cdots\boldsymbol{\Lambda}_{kT}\right),\ j=1,\ldots,M,\ k=1,\ldots,K, \tag{16}$$

where $\boldsymbol{\Psi}=\operatorname{diag}(\psi_1,\ldots,\psi_M), \boldsymbol{\Lambda}_{kT}=\operatorname{diag}\left(\lambda_{f(1,t),k},\ldots,\lambda_{f(M,t),k}\right), t=1,\ldots,T.$

The Gibbs sampler then involves the following steps in each MCMC iteration:

- update $\boldsymbol{\beta}_k : \boldsymbol{\beta}_k \mid \cdot \sim \mathcal{MVN}(\mu_k, \boldsymbol{\Sigma}_k), \quad \mu_k = \frac{N_k}{\sigma_k^2}\boldsymbol{\Sigma}_k\hat{\boldsymbol{\beta}}_k, \quad \boldsymbol{\Sigma}_k = \frac{\sigma_k^2}{N_k}\left(\boldsymbol{D}_k + (\phi_k\boldsymbol{\Psi}\boldsymbol{\Lambda}_{k1}\cdots\boldsymbol{\Lambda}_{kT})^{-1}\right)^{-1},$

where D_k is the LD-matrix for population k, $\hat{\boldsymbol{\beta}}_k$ is the marginal least squares estimates obtained from GWAS summary statistics. To avoid the numerical issue caused by colinearity between SNPs, we restrict $(\phi_k \boldsymbol{\Psi} \boldsymbol{\Lambda}_{k1} \cdots \boldsymbol{\Lambda}_{kT})^{-1} \geq 1$.

- update $\sigma_k^2 : \sigma_k^2 \mid \cdot \sim \mathcal{IG}\left(\frac{N_k+M_k}{2}, \frac{N_k}{2}\left[1 - 2\boldsymbol{\beta}_k^T \hat{\boldsymbol{\beta}}_k + \boldsymbol{\beta}_k^T \left(\boldsymbol{D}_k + \phi_k \boldsymbol{\Psi} \boldsymbol{\Lambda}_{k1} \cdots \boldsymbol{\Lambda}_{kT}\right) \boldsymbol{\beta}_k\right]\right)$
- update $\phi_k : \phi_k \mid \cdot \sim \mathcal{IG}\left(\frac{M_k+2}{2}, \frac{\boldsymbol{\beta}_k^T (\boldsymbol{\Psi} \boldsymbol{\Lambda}_{k1} \cdots \boldsymbol{\Lambda}_{kT})^{-1} \boldsymbol{\beta}_k N_k}{2\sigma_k^2} + \frac{1}{\nu}\right)$
- update $\nu_k : \nu_k \mid \cdot \sim \mathcal{IG}\left(1, \frac{1}{\phi_k} + 1\right)$
- update $\psi_j : \psi_j \mid \cdot \sim \mathcal{IG}\left(\frac{k_j+1}{2}, \sum_{k=1}^{K} \frac{\beta_{jk}^2 N_k}{2\sigma_k^2 \phi_k \prod_{t=1}^{T} \lambda_{f(j,t),k}} + \frac{1}{c_j}\right)$

  where $k_j = 1$ if SNP j exists only in one population and r if it exists in r populations included.
- update $c_j : c_j \mid \cdot \sim \mathcal{IG}\left(1, \frac{1}{\psi_j} + 1\right)$
- update $\lambda_{a_t,k} : \lambda_{a_t,k} \mid \cdot \sim \mathcal{IG}\left(\frac{s_{a_t}+1}{2}, \frac{1}{2\phi_k} \sum_{j \in l(a_t,k)} \frac{\beta_{jk}^2 N_k}{\prod_{t \neq t} \lambda_{f(j,t),k} \psi_j \sigma_k^2} + \frac{1}{t_{a,k}}\right)$

  where s_{a_t} is the number of predictors in category a_t, $l(a_t, k) = \{j \in \{1, \ldots, M\} : \lambda_{f(j,t),k} = \lambda_{a_t,k}\}$ is the set of predictors belonging to category a_t.
- update $t_{a_t,k} \mid \cdot \sim \mathcal{IG}\left(1, \frac{1}{\lambda_{a_t,k}} + 1\right)$

3 Combining multiple PRS with GWAS summary statistics

3.1 Sufficient statistics for least squares estimator of linear combination weights

Consider the linear combination problem for K centered population-specific PRS using the individual-level validation data $\left(\boldsymbol{X}_1^{(v)}, \boldsymbol{Y}_1^{(v)}\right)$ with sample size $N_1^{(v)}$:

$$\boldsymbol{Y}_1^{(v)} \sim \boldsymbol{PRS}^{(v)} \boldsymbol{w}. \tag{17}$$

Here, superscript v highlights the fact that phenotypes and PRS in this regression exercise need to be obtained from a validation dataset that is different from any data used for GWAS and PRS training. $\boldsymbol{Y}_1^{(v)}$ is the phenotype vector and $\boldsymbol{PRS}^{(v)}$ is the $N_1^{(v)} \times K$ matrix of K population-specific scores in this sample. Further, $\boldsymbol{PRS}^{(v)}$ can be denoted as $\boldsymbol{PRS}^{(v)} = \boldsymbol{X}_1^{(v)} \boldsymbol{b}$ where $\boldsymbol{X}_1^{(v)}$ is the $N_1^{(v)} \times M$ genotype matrix and $\boldsymbol{b}$ is the $M \times K$ matrix for SNP effects, $\boldsymbol{w} = [w_1, \ldots, w_K]^T$ is a K-dimensional linear combination weights vector.For simplicity, we assume $\boldsymbol{Y}_1^{(v)}$ is centered, $\boldsymbol{X}_1^{(v)}$ is standardized, and $\boldsymbol{b}$ quantifies standardized SNP effects.

Next, we showed the The least squares estimator for $\boldsymbol{w}$ is

$$\begin{aligned} \widehat{\boldsymbol{w}} &= \left[\boldsymbol{PRS}^{(v)T} \boldsymbol{PRS}^{(v)}\right]^{-1} \boldsymbol{PRS}^{(v)T} \boldsymbol{Y}_1^{(v)} \\ &= \left[\boldsymbol{b}^T \boldsymbol{X}_1^{(v)T} \boldsymbol{X}_1^{(v)} \boldsymbol{b}\right]^{-1} \boldsymbol{b}^T \boldsymbol{X}_1^{(v)T} \boldsymbol{Y}_1^{(v)}. \end{aligned} \tag{18}$$

This indicates that $\boldsymbol{b}, \boldsymbol{X}_1^{(v)T}\boldsymbol{X}_1^{(v)}$, and $\boldsymbol{X}_1^{(v)T}\boldsymbol{Y}_1^{(v)}$ are sufficient statistics for $\widehat{\boldsymbol{w}}$, where $\boldsymbol{b}$ is obtained from the PRS training procedure, $\boldsymbol{X}_1^{(v)T}\boldsymbol{X}_1^{(v)}$ is from in-sample LD matrix, and $\boldsymbol{X}_1^{(v)T}\boldsymbol{Y}_1^{(v)}$ can be obtained from the summary statistics of the validation sample. When the in-sample LD information is not available, we use LD matrix from the reference panel $\frac{\boldsymbol{X}^{(ref)T}\boldsymbol{X}^{(ref)}}{N^{(ref)}}$ as replacement. Then we have

$$\begin{aligned}
\widehat{\boldsymbol{w}} &= \left[\boldsymbol{b}^T\boldsymbol{X}_1^{(v)T}\boldsymbol{X}_1^{(v)}\boldsymbol{b}\right]^{-1}\boldsymbol{b}^T\boldsymbol{X}_1^{(v)T}\boldsymbol{Y}_1^{(v)} \\
&\approx \left[\frac{N_1^{(v)}}{N^{(ref)}}\boldsymbol{b}^T\boldsymbol{X}^{(ref)T}\boldsymbol{X}^{(ref)}\boldsymbol{b}\right]^{-1}\boldsymbol{b}^T\boldsymbol{X}_1^{(v)T}\boldsymbol{Y}_1^{(v)} \\
&= \frac{N^{(ref)}}{N_1^{(v)}}\left[\boldsymbol{PRS}^{(ref)T}\boldsymbol{PRS}^{(ref)}\right]^{-\mathbf{1}}\boldsymbol{b}^T\boldsymbol{X}_1^{(v)T}\boldsymbol{Y}_1^{(v)},
\end{aligned} \tag{19}$$

where $N^{(ref)}$ and $\boldsymbol{PRS}^{(ref)}$ denote the sample size and PRS matrix in the reference panel. Taken together, this shows that in order to obtain $\widehat{\boldsymbol{w}}$, we only need the LD reference and summary statistics from a validation sample.

3.2 Derivation for subsampling GWAS summary statistics from training and validation sets

Consider the phenotype-genotype model:

$$Y_i = \boldsymbol{X_i}\boldsymbol{\beta} + \epsilon_i, \tag{20}$$

where Y_i is the standardized phenotype with mean 0 and variance 1 for individual i, $\boldsymbol{X_i}$ is a $1\times M$ standardized genotype matrix, and ϵ_i is the error term, $\boldsymbol{\beta}$ is a p-dimensional effect sizes vector. Note that the subscript i in section 3.2 denotes the individual rather than the population.

Here, we consider X_i and Y_i as random and *i.i.d.* distributed (*i.e.,* $Y_1, \dots, Y_N \overset{i.i.d}{\sim} Y_1 \in \mathbb{R}$, $\boldsymbol{X}_1, \dots, \boldsymbol{X}_N \overset{i.i.d}{\sim} \boldsymbol{X}_1 \in \mathbb{R}^{M\times 1}$). We denote $\boldsymbol{Y} = (Y_1, \dots, Y_N)^T$ as a N-dimensional phenotype vector and $\boldsymbol{X} = (\boldsymbol{X}_1^T, \dots, \boldsymbol{X}_N^T)^T$ as a $N \times M$ standardized genotype matrix.

The standard approach the process for model validation technique involves first randomly sampling a subset of $N - N^{(v)}$ individuals from full sample $(\boldsymbol{X}, \boldsymbol{Y})$ as the training data $(\boldsymbol{X}^{(tr)}, \boldsymbol{Y}^{(tr)})$, and use the remaining $N^{(v)}$ individuals as the validation data $(\boldsymbol{X}^{(v)}, \boldsymbol{Y}^{(v)})$,

The GWAS sample size is large and hence by the central limit theorem, we have approximately

$$\begin{aligned}
&\boldsymbol{X}^T\boldsymbol{Y} \sim \mathcal{N}(N\mathbb{E}[\boldsymbol{X}_1^T Y_1], N\,\mathrm{Var}[\boldsymbol{X}_1^T Y_1]) \\
&\boldsymbol{X}^{(tr)T}\boldsymbol{Y}^{(tr)} \sim \mathcal{N}((N - N_1^{(v)})\mathbb{E}[\boldsymbol{X}_1^T Y_1], (N - N_1^{(v)})\,\mathrm{Var}[\boldsymbol{X}_1^T Y_1]).
\end{aligned} \tag{21}$$

The covariance between $\boldsymbol{X}^T\boldsymbol{Y}$ and $\boldsymbol{X}^{(tr)T}\boldsymbol{Y}^{(tr)}$ is

$$\begin{aligned}
\mathrm{Cov}(\boldsymbol{X}^T\boldsymbol{Y}, \boldsymbol{X}^{(tr)T}\boldsymbol{Y}^{(tr)}) &= \mathrm{Cov}(\boldsymbol{X}^{(tr)T}\boldsymbol{Y}^{(tr)} + \boldsymbol{X}^{(v)T}\boldsymbol{Y}^{(v)}, \boldsymbol{X}^{(tr)T}\boldsymbol{Y}^{(tr)}) \\
&= \mathrm{Var}(\boldsymbol{X}^{(tr)T}\boldsymbol{Y}^{(tr)}) \\
&= (N - N^{(v)})\,\mathrm{Var}[\boldsymbol{X}_1^T Y_1].
\end{aligned} \tag{22}$$

Here, we use the formula for the conditional distribution of two multivariate normal random vectors: if $\boldsymbol{A} \sim \mathcal{N}(\boldsymbol{\mu_A}, \boldsymbol{\Sigma_A})$, $\boldsymbol{B} \sim \mathcal{N}(\boldsymbol{\mu_B}, \boldsymbol{\Sigma_B})$, and $\text{Cov}(\boldsymbol{A}, \boldsymbol{B}) = \boldsymbol{\Sigma_{AB}}$, we have the distribution of $\boldsymbol{A}|\boldsymbol{B}$ following a multivariate normal distribution with mean and covariance matrix.

$$\begin{aligned} \mathbb{E}[\boldsymbol{A} \mid \boldsymbol{B} = \boldsymbol{b}] &= \boldsymbol{\mu_A} + \boldsymbol{\Sigma_{AB}} \boldsymbol{\Sigma_B}^{-1} (\boldsymbol{b} - \boldsymbol{\mu_B}) \\ \text{Var}[\boldsymbol{A} \mid \boldsymbol{B} = \boldsymbol{b}] &= \boldsymbol{\Sigma_A} - \boldsymbol{\Sigma_{AB}} \boldsymbol{\Sigma_B}^{-1} \boldsymbol{\Sigma_{AB}}. \end{aligned} \tag{23}$$

Thus, we have

$$\begin{aligned} \mathbb{E}[\boldsymbol{X}^{(tr)T}\boldsymbol{Y}^{(tr)} \mid \boldsymbol{X}^T\boldsymbol{Y} = \boldsymbol{x}^T\boldsymbol{y}] &= (N - N^{(v)})\mathbb{E}[\boldsymbol{X}_1^T Y_1] + \frac{N - N^{(v)}}{N} \left(\boldsymbol{x}^T\boldsymbol{y} - N\mathbb{E}[\boldsymbol{X}_1^T Y_1]\right) \\ \text{Var}[\boldsymbol{X}^{(tr)T}\boldsymbol{Y}^{(tr)} \mid \boldsymbol{X}^T\boldsymbol{Y} = \boldsymbol{x}^T\boldsymbol{y}] &= (N - N^{(v)}) \text{Var}[\boldsymbol{X}_1^T Y_1] - \frac{N - N^{(v)}}{N}(N - N^{(v)}) \text{Var}[\boldsymbol{X}_1^T Y_1] \\ &= \frac{(N - N^{(v)})N^{(v)}}{N} \text{Var}[\boldsymbol{X}_1^T Y_1]. \end{aligned} \tag{24}$$

For the conditional expectation, we plug in the estimator $\boldsymbol{x}^T\boldsymbol{y}$ for $N\mathbb{E}[\boldsymbol{X}_1^T Y_1]$, the estimator for the conditional expectation is

$$\widehat{\mathbb{E}[\boldsymbol{X}^{(tr)T}\boldsymbol{Y}^{(tr)} \mid \boldsymbol{X}}^T\boldsymbol{Y} = \boldsymbol{x}^T\boldsymbol{y}] = \frac{N - N_1^{(v)}}{N} \boldsymbol{x}^T\boldsymbol{y} \tag{25}$$

For notation purpose, we define the conditional variance as

$$\text{Var}[\boldsymbol{X}_1^T Y_1] := \boldsymbol{\Sigma} \in \mathbb{R}^{M \times M} \tag{26}$$

The diagonal term $\boldsymbol{\Sigma_{jj}}$ of $\boldsymbol{\Sigma}$ is

$$\begin{aligned} \boldsymbol{\Sigma_{jj}} &= \text{Var}[X_{1j} Y_1] \\ &= \mathbb{E}[X_{1j}^2 Y_1^2] - \mathbb{E}[X_{1j} Y_1]^2 \\ &= \mathbb{E}[X_{1j}^2]\mathbb{E}[Y_1^2] + \text{Cov}(X_{1j}^2, Y_1^2) - [\mathbb{E}[X_{1j}]\mathbb{E}[Y_1] + \text{Cov}(X_{1j}, Y_1)]^2 \\ &\approx \mathbb{E}[X_{1j}^2] \\ &= 1 \end{aligned} \tag{27}$$

The off-diagonal term $\boldsymbol{\Sigma_{jk}}$ of $\boldsymbol{\Sigma}, j \neq k$ is

$$\begin{aligned} \boldsymbol{\Sigma_{jk}} &= \text{Cov}[X_{1j} Y_1, X_{1k} Y_1] \\ &= \mathbb{E}[X_{1j} X_{1k} Y_1^2] - \mathbb{E}[X_{1j} Y_1]\mathbb{E}[X_{1k} Y_1] \\ &= \mathbb{E}[X_{1j} X_{1k}]\mathbb{E}[Y_1^2] + \text{Cov}(X_{1j} X_{1k}, Y_1^2) - [\mathbb{E}[X_{1j}]\mathbb{E}[Y_1] + \text{Cov}(X_{1j}, Y_1)][\mathbb{E}[X_{1k}]\mathbb{E}[Y_1] + \text{Cov}(X_{1k}, Y_1)] \\ &\approx \mathbb{E}[X_{1j} X_{1k}] \end{aligned} \tag{28}$$

It turns out that the $\boldsymbol{\Sigma}$ is exactly the LD matrix constructed using the standardized genotypes. Thus, we obtain the estimator for the conditional variance as

$$\widehat{\text{Var}[\boldsymbol{X}^{(tr)T}\boldsymbol{Y}^{(tr)} \mid \boldsymbol{X}}^T\boldsymbol{Y} = \boldsymbol{x}^T\boldsymbol{y}] = \frac{(N - N^{(v)})N^{(v)}}{N} \hat{\boldsymbol{\Sigma}}, \tag{29}$$

where $\hat{\boldsymbol{\Sigma}} = \frac{\boldsymbol{X}^{(ref)T}\boldsymbol{X}^{(ref)}}{N^{(ref)}}$ is obtained from the reference panel, $\boldsymbol{X}^{(ref)}$ is a $N^{(ref)} \times M$ standardized genotype matrix.

In conclusion, we have

$$\boldsymbol{X}^{(tr)T}\boldsymbol{Y}^{(tr)} \mid \boldsymbol{X^T Y} = \boldsymbol{x^T y} \sim \mathcal{N}\left(\frac{(N - N^{(v)})}{N}\boldsymbol{x}^T\boldsymbol{y}, \frac{(N - N^{(v)})N^{(v)}}{N}\hat{\boldsymbol{\Sigma}}\right) \tag{30}$$

Thus, we subsample the summary statistics for training set given full summary statistics $\boldsymbol{X^T Y}$ by

$$\frac{\boldsymbol{X}^{(tr)T}\boldsymbol{Y}_1^{(tr)}}{N - N^{(v)}} \mid \boldsymbol{X^T Y} = \frac{\boldsymbol{X}^T\boldsymbol{Y}}{N} + \left(\frac{N^{(v)}}{(N - N^{(v)})N}\right)^{\frac{1}{2}} \frac{\boldsymbol{X}^{(ref)T}}{\sqrt{N^{(ref)}}}\boldsymbol{g} \tag{31}$$

where $\boldsymbol{g}$ is a $N^{(ref)}$-dimensional vector with elements drawn from a standard normal distribution.

3.3 Dealing with tuning parameters in the PRS model

If there are tuning parameters in the PRS model, we use the correlation R between phenotype and linearly combined PRS in the validation set to select the optimal tuning parameter, as well as to estimate the linear combination weights.

Followed the notation above, suppose there are tuning parameters γ in the PRS model, consider the linear combination problem for K centered PRS using the individual-level validation data:

$$\boldsymbol{Y}_1^{(v)} \sim \boldsymbol{PRS}_{\gamma}^{(v)}\boldsymbol{w}_{\gamma} \tag{32}$$

where $\boldsymbol{PRS}_{\gamma}^{(v)} = \boldsymbol{X}_1^{(v)}\boldsymbol{b}_{\gamma} = \left[\boldsymbol{PRS}_{1,\gamma}^{(v)}, \dots \boldsymbol{PRS}_{k,\gamma}^{(v)}, \dots, \boldsymbol{PRS}_{K,\gamma}^{(v)}\right]$ is a $N_1^{(v)} \times K$ centered PRS matrix with respect to to the tuning parameter γ in the PRS model, $\boldsymbol{w}_{\gamma}$ is a K-dimensional linear combination weights vector.

The least squares estimator for $\boldsymbol{w}_{\gamma}$ is

$$\hat{\boldsymbol{w}_{\gamma}} \approx \frac{N^{(ref)}}{N_1^{(v)}}\left[\boldsymbol{PRS}_{\gamma}^{(ref)T}\boldsymbol{PRS}_{\gamma}^{(ref)}\right]^{-1}\boldsymbol{b_{\gamma}^T}\boldsymbol{X}_1^{(v)T}\boldsymbol{Y}_1^{(v)} \tag{33}$$

where $\boldsymbol{PRS}_{\gamma}^{(ref)} = \boldsymbol{X}^{(ref)}\boldsymbol{b}_{\gamma}$ is $N^{(ref)} \times K$ PRS matrix in the reference panel.

The correlation R_{γ} between the linearly combined PRS and phenotype in the validation set with respect to to the estimated weights $\hat{\boldsymbol{w}_{\gamma}}$ is :

$$\begin{aligned} R_{\gamma} &= \frac{\hat{\boldsymbol{w}_{\gamma}}^T\boldsymbol{b}_{\gamma}^T\boldsymbol{X}_1^{(v)T}\boldsymbol{Y}_1^{(v)}/N_1^{(v)}}{\left(\hat{\boldsymbol{w}_{\gamma}}^T\boldsymbol{b}_{\gamma}^T\boldsymbol{X}_1^{(v)T}\boldsymbol{X}_1^{(v)}\boldsymbol{b}_{\gamma}\hat{\boldsymbol{w}_{\gamma}}/N_1^{(v)}\right)^{1/2}} \\ &\approx \frac{\hat{\boldsymbol{w}_{\gamma}}^T\boldsymbol{b}_{\gamma}^T\boldsymbol{X}_1^{(v)T}\boldsymbol{Y}_1^{(v)}/N_1^{(v)}}{\left(\hat{\boldsymbol{w}_{\gamma}}^T\boldsymbol{b}_{\gamma}^T\boldsymbol{X}_1^{(ref)T}\boldsymbol{X}_1^{(ref)}\boldsymbol{b}_{\gamma}\hat{\boldsymbol{w}_{\gamma}}/N^{(ref)}\right)^{1/2}} \\ &= \frac{\hat{\boldsymbol{w}_{\gamma}}^T\boldsymbol{b}_{\gamma}^T\boldsymbol{X}_1^{(v)T}\boldsymbol{Y}_1^{(v)}/N_1^{(v)}}{\left(\hat{\boldsymbol{w}_{\gamma}}^T\boldsymbol{PRS}_{\gamma}^{(ref)T}\boldsymbol{PRS}_{\gamma}^{(ref)}\hat{\boldsymbol{w}_{\gamma}}/N^{(ref)}\right)^{1/2}} \end{aligned} \tag{34}$$

Then we select the optimal tuning parameter $\hat{\gamma}$ as

$$\hat{\gamma} = \text{argmax}_{\gamma} R_{\gamma}, \tag{35}$$

and use the linear combination weights $\hat{\boldsymbol{w}}_{\hat{\gamma}}$ with respect to the optimal tuning parameter $\hat{\gamma}$ to linearly combine the PRS.

3.4 Grid search to handle negative least squares estimates for mixing weights

In practice, the least squares estimates for linear combination weights of a particular PRS can be negative. It may decrease the prediction accuracy of the linearly combined PRS. Thus, we provide a grid search strategy to mimic the non-negative least squares.

We pre-specify a grid of positive value for the linear combination weights

$$\boldsymbol{w} \in \boldsymbol{W} = \{(w_1, \dots, w_k, \dots, w_K) \mid \sum_{k=1}^{K} w_k = 1, w_k \geq 0 \text{ for all } k = 1, \dots, K\}. \tag{36}$$

Then, we use the formula in the above section to calculate the correlation between the linearly combined PRS and phenotype in the validation set. The linear combination weights with respect to to largest correlation, $\hat{\boldsymbol{w}}_{grid} = \text{argmax}_{\boldsymbol{w} \in \boldsymbol{W}} R_{\boldsymbol{w}}$, will be used to linearly combine the PRS.

3.5 Summary statistics-based ridge regression to combine multiple PRS

When linearly combined many PRS with multicollinearity problems, the least squares of the linear combination weights may be sub-optimal. A remedy for multicollinearity is ridge regression.

We first describe a individual-level data-based ridge regression. Consider the linear combination problem for K centered population-specific PRS using the individual-level validation data $\left(\boldsymbol{X}_1^{(v)}, \boldsymbol{Y}_1^{(v)}\right)$ with sample size $N_1^{(v)}$:

$$\boldsymbol{Y}_1^{(v)} \sim \boldsymbol{PRS}^{(v)} \boldsymbol{w}. \tag{37}$$

Here, $\boldsymbol{Y}_1^{(v)}$ is the phenotype vector and $\boldsymbol{PRS}^{(v)}$ is the $N_1^{(v)} \times K$ matrix of K population-specific scores in this sample. Further, $\boldsymbol{PRS}^{(v)}$ can be denoted as $\boldsymbol{PRS}^{(v)} = \boldsymbol{X}_1^{(v)} \boldsymbol{b}$ where $\boldsymbol{X}_1^{(v)}$ is the $N_1^{(v)} \times M$ genotype matrix and $\boldsymbol{b}$ is the $M \times K$ matrix for SNP effects, $\boldsymbol{w} = [w_1, \dots, w_K]^T$ is a K-dimensional linear combination weights vector. For simplicity, we assume $\boldsymbol{Y}_1^{(v)}$ is centered, $\boldsymbol{X}_1^{(v)}$ is standardized, and $\boldsymbol{b}$ quantifies standardized SNP effects.

The ridge regression estimator for $\boldsymbol{w}$ is

$$\widehat{\boldsymbol{w}}_{ridge,\lambda} = \underset{\boldsymbol{w} \in \mathbb{R}^K}{\text{argmin}} \|\boldsymbol{Y}_1^{(v)} - \boldsymbol{PRS}^{(v)} \boldsymbol{w}\|_2^2 + \lambda \|\boldsymbol{w}\|_2^2, \tag{38}$$

where λ is the shrinkage parameter. It has a closed-form solution:

$$\begin{aligned}\widehat{\boldsymbol{w}}_{ridge,\lambda} &= \left[\boldsymbol{b}^T\boldsymbol{X}_1^{(v)T}\boldsymbol{X}_1^{(v)}\boldsymbol{b} + \lambda\boldsymbol{I}_K\right]^{-1}\boldsymbol{b}^T\boldsymbol{X}_1^{(v)T}\boldsymbol{Y}_1^{(v)} \\ &\approx \left[\frac{N_1^{(v)}}{N^{(ref)}}\boldsymbol{PRS}^{(ref)T}\boldsymbol{PRS}^{(ref)T} + \lambda\boldsymbol{I}_K\right]^{-1}\boldsymbol{b}^T\boldsymbol{X}_1^{(v)T}\boldsymbol{Y}_1^{(v)}.\end{aligned} \tag{39}$$

Here, $\boldsymbol{PRS}^{(ref)} = \boldsymbol{X}^{(ref)}\boldsymbol{b}$, $\boldsymbol{X}^{(ref)}$ is a $N^{(ref)} \times M$ standardized genotype matrix in reference panel. A common practice to obtain the ridge regression estimator is to use two disjoint validation set, one to select the optimal tuning parameter λ and the other to estimate $\boldsymbol{w}$ with the selected tuning parameter.

Next, we proposed a summary statistics-based ridge regression for combining multiple PRS. Given the GWAS summary statistics with sample size N_1, we subsample GWAS summary statistics for the training set $\boldsymbol{X}_1^{(tr)T}\boldsymbol{Y}^{(tr)}$ with sample size $N_1 - N_1^{(v)}$, and for the validation set $\boldsymbol{X}_1^{(v)T}\boldsymbol{Y}_1^{(v)}$ with sample size $N_1^{(v)}$. We further use to $\boldsymbol{X}_1^{(v)T}\boldsymbol{Y}_1^{(v)}$ to subsample GWAS for two disjoint validation sets: $\boldsymbol{X}_1^{(v1)T}\boldsymbol{Y}_1^{(v1)}$ for validation set 1 with sample size $N_1^{(v_1)}$ and $\boldsymbol{X}_1^{(v_2)T}\boldsymbol{Y}_1^{(v_2)}$ for validation set 2 with sample size $N_1^{(v_2)} = N_1^{(v)} - N_1^{(v_1)}$.

We first apply the PRS method using $\boldsymbol{X}_1^{(tr)T}\boldsymbol{Y}^{(tr)}$ as training data to obatin SNP effects $\boldsymbol{b}$. Next, we obtain grid of the ridge regression estimate $\widehat{\boldsymbol{w}}_{ridge,\lambda}$ using $\boldsymbol{X}_1^{(v1)T}\boldsymbol{Y}_1^{(v1)}$ from validation set 1 and grid of value $\lambda \in \Lambda$. The λ with respect to the largest correlation between phenotype and linearly combined PRS in validation set 1 will be used:

$$\hat{\lambda} = \text{argmax}_{\lambda\in\boldsymbol{\Lambda}}\frac{\widehat{\boldsymbol{w}}_{ridge,\lambda}^T\boldsymbol{b}^T\boldsymbol{X}_1^{(v1)T}\boldsymbol{Y}_1^{(v1)}/N_1^{(v_1)}}{\left(\widehat{\boldsymbol{w}}_{ridge,\lambda}^T\boldsymbol{PRS}^{(ref)T}\boldsymbol{PRS}^{(ref)}\widehat{\boldsymbol{w}}_{ridge,\lambda}/N^{(ref)}\right)^{1/2}}, \tag{40}$$

where $\boldsymbol{PRS}^{(ref)} = \boldsymbol{X}_1^{(ref)}\boldsymbol{b}$, $\boldsymbol{X}_1^{(ref)}$ is the $N^{(ref)} \times M$ standard genotype matrix in target population reference panel

The final ridge regression-based linear combination weights is estimated in validation set 2 using the formula below:

$$\widehat{\boldsymbol{w}}_{ridge,\hat{\lambda}} = \left[\frac{N_1^{(v_2)}}{N^{(ref)}}\boldsymbol{PRS}^{(ref)T}\boldsymbol{PRS}^{(ref)} + \hat{\lambda}\boldsymbol{I}_K\right]^{-1}\boldsymbol{b}^T\boldsymbol{X}_1^{(v_2)T}\boldsymbol{Y}_1^{(v_2)}. \tag{41}$$

where $\boldsymbol{PRS}^{(ref)} = \boldsymbol{X}^{(ref)}\boldsymbol{b}$, $\boldsymbol{X}^{(ref)}$ is a $N^{(ref)} \times M$ standardized genotype matrix in reference panel.

To avoid overfitting, we recommend using distinct reference panel in summary statistics sampling, PRS model training, ridge regression hyperparameter λ selection, and linear combination weights estimation.

3.6 Transforming allele count scale SNP effects into standardized scale

For simplicity, we assume that the genotype matrix is standardized, thus the SNP effects should be on standardized allele scale. Since many PRS method outputs the allele count SNP effects , we use allele frequency from the target population to transform the allele count SNP effects to the standardized effects. The k-th column $\boldsymbol{b}_k$ in $M \times K$ SNP effects matrix $\boldsymbol{b} = [\boldsymbol{b}_1, \ldots \boldsymbol{b}_K]$ is calculated by

$$\boldsymbol{b}_k = \frac{\boldsymbol{b}_k^{(\text{allele})}}{\sqrt{2\boldsymbol{f}_1\left(1-\boldsymbol{f}_1\right)}}, \tag{42}$$

where $\boldsymbol{b}_k^{(\text{allele})}$ is the allele count SNP effects for M SNPs in k-th population, $\boldsymbol{f}_1$ is the M-dimensional target population allele frequency vector. When the in-sample allele frequency is not available, we estimated it from the target population reference panel.

3.7 GWAS summary statistics-based cross-validation

Suppose we divide the full GWAS sample $(\boldsymbol{X}_1, \boldsymbol{Y}_1)$ into a training set $\left(\boldsymbol{X}_1^{(tr)}, \boldsymbol{Y}_1^{(tr)}\right)$ with $N_1 - N_1^{(v)}$ individuals, and a validation set $\left(X_1^{(v)}, Y_1^{(v)}\right)$ with $N_1^{(v)}$ individuals. Given the association z-scores $\left(\frac{\boldsymbol{X}_1^T\boldsymbol{Y}_1}{\sqrt{N_1}}\right)$ from GWAS summary statistics and genotype data from the reference panel, association summary statistics based on training and validation sets can be sampled as:

$$\begin{aligned}\frac{\boldsymbol{X}_1^{(tr)T}\boldsymbol{Y}_1^{(tr)}}{N_1 - N_1^{(v)}} &= \frac{\boldsymbol{X}_1^T\boldsymbol{Y}_1}{N_1} + \left(\frac{N_1^{(v)}}{N_1(N_1 - N_1^{(v)})}\right)^{\frac{1}{2}} \frac{\boldsymbol{X}^{(ref)T}}{\sqrt{N^{(ref)}}}\boldsymbol{g} \\ \frac{\boldsymbol{X}_1^{(v)T}\boldsymbol{Y}_1^{(v)}}{N_1^{(v)}} &= \frac{\boldsymbol{X}_1^T\boldsymbol{Y}_1 - \boldsymbol{X}_1^{(tr)T}\boldsymbol{Y}_1^{(tr)}}{N_1^{(v)}}\end{aligned} \quad (43)$$

where $\boldsymbol{X}^{(ref)}$ is a $N^{(ref)} \times M$ standardized genotype matrix from the reference panel for the target population, $N^{(ref)}$ is the sample size of the reference panel, $\boldsymbol{g}$ is a $N^{(ref)}$-dimensional vector with elements drawn from a standard normal distribution.

To perform P-folds cross-validation, we first uses the above formula to sample $\boldsymbol{X}_{1,p}^T\boldsymbol{Y}_{1,p}, p = 1, \dots P-1$ from P independent subset with sample size $\left[\frac{N_1}{P}\right]$ and obtain the GWAS summary statistics from training and validation sets in fold p as:

$$\begin{aligned}\frac{\boldsymbol{X}_{1,p}^{(tr)T}\boldsymbol{Y}_{1,p}^{(tr)}}{N_1 - \left[\frac{N_1}{P}\right]} &= \frac{\boldsymbol{X}_1^T\boldsymbol{Y}_1 - \boldsymbol{X}_{1,p}^T\boldsymbol{Y}_{1,p}}{N_1 - \left[\frac{N_1}{P}\right]} \\ \frac{\boldsymbol{X}_{1,p}^{(v)T}\boldsymbol{Y}_{1,p}^{(v)}}{\left[\frac{N_1}{P}\right]} &= \frac{\boldsymbol{X}_{1,p}^T\boldsymbol{Y}_{1,p}}{\left[\frac{N_1}{P}\right]},\end{aligned} \quad (44)$$

and estimate the linear combination weights in each fold.

3.8 Regarding the marginal linear assumptions

X-Wing assumes a marginal linear regression between the phenotype and SNP. In practice, many of the GWAS are performed using the mixed model. Mathematically, the GWAS association results using the linear mixed model is equivalent to the results using the marginal linear model on phenotypic residual after adjusting for best linear unbiased prediction (BLUP). This phenotypic residual can be considered the phenotype after adjusting for the sample relatedness (genetic relationship matrix). In our analysis, the BBJ GWAS are performed using BOLT-LMM. BOLT-LMM utilized a two-step approach to conduct the linear mixed model GWAS: "Our algorithm fits a Gaussian mixture model of SNP using a fast variational approximation to compute approximate phenotypic residuals and tests the residuals for association with candidate markers via a

retrospective score statistic”. Therefore, the mixed model GWAS from BOLT-LMM is from the marginal linear regression between the phenotypic residuals and the SNP. To summarize, most current GWAS (such as BBJ used in this study) essentially comes from a marginal linear model with phenotypic residuals as outcome and SNP as predictor. Therefore, our marginal effects assumption is valid, and we don’t expect it will influence the PRS model performance. And our repeated learning approach can be considered as splitting on this phenotypic residual instead of the raw phenotype that is correlated among correlated individuals. Therefore, applying the summary statistics-based splitting using summary statistics that have already been accounted for sample relatedness should not cause problems.

4 Implementation of other methods

XPASS XPASS[1] is an empirical Bayes-based PRS framework that leverages genetic correlation for cross-population polygenic prediction. In our paper, XPASS is used to compute heritability and cross-population genetic covariance (correlation), and estimate the SNP posterior effects used to calculate PRS. We used population-matched 1000 Genomes Project data as the reference panel. Five principal components of genotypes in reference panel were used as covariate files as suggested by the software. We estimated the global genetic correlation using genome-wide SNPs. We also created two SNP sets: SNPs inside and outside significant genome regions identified by X-Wing, and computed cross-population genetic correlation using GWAS summary statistics restricted to the two SNP sets separately. Standard errors of genetic parameters (heritability, genetic covariance, and genetic correlation) were estimated using block-wise jackknife method. For PRS construction, we obtained the posterior effects for each population to generate population-specific PRS. Although XPASS did not propose to linearly combine PRS, we applied the linear combination to XPASS-derived PRS for a fair comparison.

PESCA As suggested by PESCA paper[2], we pruned SNPs such that correlation between SNPs does not exceed 0.95 in the population-matched 1000 Genomes Project data. We used ldetect[3] to produce LD blocks that are approximately independent in both populations. 14,630 SNPs and 31 independent LD blocks on chromosome 22 were used in simulations; Approximately 500,000 SNPs and 1,368 independent LD blocks were used in GWAS analysis of 31 complex traits. Maximum number of EM iterations to estimate genome-wide prior probability was set as `100` with flag `-max_iter`. Number of independent MCMC chains in estimating posteriors was set as `20` with flag `-max_iter_post`. Number of burn-ins and samples for the MCMC were both set as `5000` by default with flags `-nburn` and `-nsample`, respectively. SNPs with posterior probability larger than 0.95 were identified as shared causal SNPs across populations. To compare findings between X-Wing and PESCA, we extended SNPs identified by PESCA into regions with equal size, such that the aggregated size was the same as that of X-Wing.

PolyFun-pred PolyFun-pred[4] uses SNP effects estimated from functionally informed fine-mapping to calculate PRS. We downloaded the pre-computed PRS coefficients for PolyFun-pred and used these coefficients to generate PRS. After overlapping with the trait list for the GWAS summary statistics we used, there are 16 traits for East Asians and 11 traits for Americans left.

Polypred+ Polypred+[4] linearly combines the effect sizes of BOLT-LMM, PolyFun-pred (trained using European training data), and BOLT-LMM-pop (trained using non-European training data from the target population). We downloaded the pre-computed PRS coefficients for BOLT-LMM-UKB, PolyFun-pred, and BOLT-LMM-BBJ, and then used these coefficients to generate PRS. After overlapping with the trait list for the GWAS summary statistics we used, there are 16 traits for East Asians left.

PRS-CSx PRS-CSx[5] is a Bayesian cross-population PRS framework with shared continuous shrinkage across the different populations. We ran PRS-CSx using the default population-matched 1000 Genomes Project data as the reference panel. We used hyperparameter `a=0.5` and `b=0.5` in PRS-CSx, which is exactly the horseshoe prior, for a fair comparison with our method. This choice of the value of `a` and `b` performed almost the same as the default for PRS-CSx (`a=1`, `b=0.5`) given the observation in Table S9 of PRS-CSx paper[5]. The global shrinkage parameter was obtained from full Bayesian approach (automatically estimated from data) or model-tuning strategy (value among $\{10^{-6}, 10^{-4}, 10^{-2}, 1\}$ that gives the largest R^2 in the validation set will be used).

5 Simulation details

We conducted simulations to compare the predictive accuracy (measured by R2) of X-Wing with two existing methods, PRS-CSx and XPASS. We used HapGen2-simulated genotypes for Europeans and East Asians released in Zhang et al.1, which contains 956,041 Hapmap3 SNPs after quality control. We randomly selected 50 genomic segments, each spanning 1,000 SNPs. We assumed that SNPs in these correlated signal regions are causal variants shared between the two populations and the effect correlation was set to be 1. The heritability was set to be 0.5 in both populations. We performed GWAS on 50,000 Europeans and 10,000 East Asians. We used the top 100 positive correlated regions as local genetic correlation annotation in X-Wing and repeated the simulation 20 times. For both European PRS and linearly combined PRS, X-Wing outperforms PRS-CSx and XPASS across all replicates (**Supplementary Figure 19**).

6 Computational cost

X-Wing by default runs analysis on each chromosome and estimates the posterior effects for each population in parallel. The computational demands of X-Wing depend on the number of SNPs in GWAS summary statistics. For our analysis using UKBB and BBJ summary statistics as discovery GWAS and UKBB East

Asian samples as test data, it takes about 1.5 hours to finish estimating posterior effects in chromosome 1 and about 10 minutes in chromosome 22 with 2000 MCMC iterations (default), using single thread in Intel Xeon Gold E5-4620 processor (2.60 GHz).

We further compared the computation cost and requirements between X-Wing, PRS-CSx, and XPASS using the same machine. XPASS does not allow parallel computation but takes the shortest time to finish the computing for all 22 chromosomes. Both PRS-CSx and X-Wing allow parallel computing over chromosomes. For the longest chromosome (chromosome1), PRS-CSx takes about 1 hour to finish the computation and has similar memory usage as X-Wing **(Supplementary Data 25**). Since X-Wing requires estimating the posterior effects for each population separately, the number of parallel computing jobs of X-Wing is K times of PRS-CSx, where K is the number of GWAS summary statistics included.

7 Supplementary References

[1] Cai, M. *et al.* A unified framework for cross-population trait prediction by leveraging the genetic correlation of polygenic traits. *The American Journal of Human Genetics* **108**, 632–655 (2021).

[2] Shi, H. *et al.* Localizing components of shared transethnic genetic architecture of complex traits from gwas summary data. *The American Journal of Human Genetics* **106**, 805–817 (2020).

[3] Berisa, T. & Pickrell, J. K. Approximately independent linkage disequilibrium blocks in human populations. *Bioinformatics* **32**, 283 (2016).

[4] Weissbrod, O. *et al.* Leveraging fine-mapping and multipopulation training data to improve cross-population polygenic risk scores. *Nature Genetics* 1–9 (2022).

[5] Ruan, Y. *et al.* Improving polygenic prediction in ancestrally diverse populations. *Nature Genetics* 1–8 (2022).
