# Supplementary material for: Quantifying portable genetic effects and improving cross-ancestry genetic prediction with GWAS summary statistics
Source: Nat Commun. 2023 Feb 14;14:832. doi: 10.1038/s41467-023-36544-7 (PMC9929290; doi:10.1038/s41467-023-36544-7)
Supplement: Supplementary file 3 — Description of Additional Supplementary Files [file 41467_2023_36544_MOESM3_ESM.pdf]

Description of Additional Supplementary Files

One Excel file with 25 tabs

Supplementary Data 1: Type I error rates under infinitesimal model

Supplementary Data 2: Type I error rates under the heritability enrichment model

Supplementary Data 3: Type I error rates under the LDAK model

Supplementary Data 4: Type I error rates for binary traits

Supplementary Data 5: Type I error rates under the infinitesimal model for whole genome SNPs

Supplementary Data 6: False positive rates under the heritability enrichment model for whole genome SNPs

Supplementary Data 7: Description of 31 anthropometric and blood panel traits

Supplementary Data 8: Number of significant regions identified by X-Wing and PESCA in UKB-BBJ analysis

Supplementary Data 9: Cross-population genetic correlations in UKB-BBJ analysis

Supplementary Data 10: Description of four lipid traits in replication datasets

Supplementary Data 11: Genetic covariance analysis in replication datasets

Supplementary Data 12: Prediction accuracy of X-Wing and PRS-CSx for 31 traits in East Asian population

Supplementary Data 13: Prediction accuracy of X-Wing and PRS-CSx European PRS using SNPs within and outside genomic regions annotated by local genetic correlations for 31 traits in East Asian population

Supplementary Data 14: Prediction accuracy of different PRS methods for 13 traits in admixed American population

Supplementary Data 15: Prediction accuracy of different PRS methods for 13 traits in African population

Supplementary Data 16: Prediction accuracy of X-Wing PRS with varying numbers of top regions for 31 traits in East Asian population

Supplementary Data 17: P-value for comparing the prediction accuracy of X-Wing PRS with varying numbers of top regions for 31 traits in East Asian population

Supplementary Data 18: Prediction accuracy of X-Wing PRS with varying numbers of top regions for 13 traits in admixed American population

Supplementary Data 19: P-value for comparing the prediction accuracy of X-Wing PRS with varying numbers of top regions for 13 traits in admixed American population

Supplementary Data 20: Prediction accuracy of X-Wing PRS with or without MHC region removed for 31 traits in East Asian population

Supplementary Data 21: Prediction accuracy of X-Wing PRS with varying upper bound of region size for 31 traits in East Asian population

Supplementary Data 22: Prediction accuracy of X-Wing and PRS-CSx PRS using tuning parameter

Supplementary Data 23: Prediction accuracy of GWAS summary statistics-based linearly combined PRS

Supplementary Data 24: Prediction accuracy of different PRS methods for type 2 diabetes in East Asians

Supplementary Data 25: Computational time and memory of X-Wing, PRS-CSx, and XPASS
