# Supplementary material for: Quantifying portable genetic effects and improving cross-ancestry genetic prediction with GWAS summary statistics
Source: Nat Commun. 2023 Feb 14;14:832. doi: 10.1038/s41467-023-36544-7 (PMC9929290; doi:10.1038/s41467-023-36544-7)
Supplement: Supplementary file 5 — Reporting Summary [file 41467_2023_36544_MOESM5_ESM.pdf]

nature portfolio

Corresponding author(s): Qiongshi Lu

Last updated by author(s): Jan 16, 2023

Reporting Summary

Nature Portfolio wishes to improve the reproducibility of the work that we publish. This form provides structure for consistency and transparency in reporting. For further information on Nature Portfolio policies, see our Editorial Policies and the Editorial Policy Checklist.

Statistics

For all statistical analyses, confirm that the following items are present in the figure legend, table legend, main text, or Methods section.

n/a	Confirmed	
☐	☒	The exact sample size (*n*) for each experimental group/condition, given as a discrete number and unit of measurement
☐	☒	A statement on whether measurements were taken from distinct samples or whether the same sample was measured repeatedly
☐	☒	The statistical test(s) used AND whether they are one- or two-sided<br>*Only common tests should be described solely by name; describe more complex techniques in the Methods section.*
☐	☒	A description of all covariates tested
☐	☒	A description of any assumptions or corrections, such as tests of normality and adjustment for multiple comparisons
☐	☒	A full description of the statistical parameters including central tendency (e.g. means) or other basic estimates (e.g. regression coefficient) AND variation (e.g. standard deviation) or associated estimates of uncertainty (e.g. confidence intervals)
☐	☒	For null hypothesis testing, the test statistic (e.g. *F*, *t*, *r*) with confidence intervals, effect sizes, degrees of freedom and *P* value noted<br>*Give P values as exact values whenever suitable.*
☐	☒	For Bayesian analysis, information on the choice of priors and Markov chain Monte Carlo settings
☐	☒	For hierarchical and complex designs, identification of the appropriate level for tests and full reporting of outcomes
☐	☒	Estimates of effect sizes (e.g. Cohen's *d*, Pearson's *r*), indicating how they were calculated

Our web collection on statistics for biologists contains articles on many of the points above.

Software and code

Policy information about availability of computer code

Data collection	The software used to simulate the genotypes (HAPGEN2) is available at:<br>https://mathgen.stats.ox.ac.uk/genetics_software/hapgen/hapgen2.html<br>No other softwares was used for data collection since we only used previously-collected data.
Data analysis	The code used for data analysis can be found in these following websites:<br>X-Wing: https://github.com/qlu-lab/X-Wing (May 26, 2022 version);<br>LDAK: https://dougspeed.com/ldak/ (v5.2);<br>PESCA: https://github.com/huwenboshi/pesca (v0.3-beta);<br>PRS-CSx: https://github.com/getian107/PRScsx (July 29, 2021 version);<br>XPASS: https://github.com/YangLabHKUST/XPASS (May 18, 2021 version);<br>The pipeline of our analysis can be found in https://github.com/qlu-lab/X-Wing/wiki/2.-Real-example-for-X%E2%80%90Wing

For manuscripts utilizing custom algorithms or software that are central to the research but not yet described in published literature, software must be made available to editors and reviewers. We strongly encourage code deposition in a community repository (e.g. GitHub). See the Nature Portfolio guidelines for submitting code & software for further information.

Data

Policy information about availability of data

All manuscripts must include a data availability statement. This statement should provide the following information, where applicable:

- Accession codes, unique identifiers, or web links for publicly available datasets
- A description of any restrictions on data availability
- For clinical datasets or third party data, please ensure that the statement adheres to our policy

The publicly available data used in this study can be found in these websites:
Independent LD block: https://bitbucket.org/nygcresearch/ldetect-data/src/master/;
LD reference panel: https://github.com/getian107/PRScsx;
1000G genotype data: https://mathgen.stats.ox.ac.uk/impute/1000GP_Phase3.html;
UKB GWAS summary statistics: http://www.nealelab.is/uk-biobank (round2 version);
BBJ GWAS summary statistics: http://jenger.riken.jp/en/result;
PAGE study GWAS summary statistics: https://www.ebi.ac.uk/gwas/publications/31217584;
GLGC GWAS summary statistics: http://www.broadinstitute.org/mpg/ pubs/lipids2010/
AGEN GWAS summary statistics: https://blog.nus.edu.sg/agen/summary-statistics/
PolyFun-pred PRS coefficients: http://data.broadinstitute.org/alkesgroup/polypred_results.

The individual-level data can be applied for from www.ukbiobank.ac.uk. The population-assignment can be downloaded through the UK Biobank portal as Return 2442: www.ukbiobank.ac.uk., https://pan.ukbb.broadinstitute.org/downloads.

The posterior SNP effect size estimates from X-Wing used in this work can be download in https://github.com/qlu-lab/X-Wing.

Human research participants

Policy information about studies involving human research participants and Sex and Gender in Research.

Reporting on sex and gender	Our study focuses on improving the statistical methodology for genetic risk prediction in ancestrally diverse populations. We did not perform sex- and gender-based analysis.
Population characteristics	We analyzed the individual-level data from UK Biobank (UKB) and GWAS summary statistics from UKB, Biobank Japan (BBJ), Population Architecture using Genomics and Epidemiology Consortium study (PAGE), Global Lipids Genetics Consortium (GLGC), and Genetic Epidemiology Network (AGEN). All these datasets have been thoroughly described in the following publications and websites:<br>1. Bycroft, C. et al. The UK Biobank resource with deep phenotyping and genomic data. Nature 562, 203-209 (2018).<br>2. Kanai, M. et al. Genetic analysis of quantitative traits in the Japanese population links cell types to complex human diseases. Nature Genetics 50, 390-400 (2018).<br>3. Vagai, A. et al. Overview of the BioBank Japan Project: study design and profile. J. Epidemiol. 27, S2-S8 (2017).<br>4. Wojcik, G.L. et al. Genetic analyses of diverse populations improves discovery for complex traits. Nature 570, 514-518 (2019).<br>5. Teslovich, T.M. et al. Biological, clinical and population relevance of 95 loci for blood lipids. Nature 466, 707-713 (2010).<br>6. Spracklen, C.N. et al. Association analyses of East Asian individuals and transancestry analyses with European
Recruitment	We analyzed the population cohort data that were recruited in previous studies.
Ethics oversight	UKB: The data collection from UKB was approved by UKB's Research Ethics Committee. Approval to use the UKB individuallevel data in this work was obtained under application 42148.<br><br>BBJ, PAGE, GLGC, and AGEN: only the publicly available GWAS summary statistics without individual information were used in this study.

Note that full information on the approval of the study protocol must also be provided in the manuscript.

Field-specific reporting

Please select the one below that is the best fit for your research. If you are not sure, read the appropriate sections before making your selection.

☒ Life sciences ☐ Behavioural & social sciences ☐ Ecological, evolutionary & environmental sciences

For a reference copy of the document with all sections, see nature.com/documents/nr-reporting-summary-flat.pdf

Life sciences study design

All studies must disclose on these points even when the disclosure is negative.

Sample size	Simulation: 50,000 individuals with genotype and phenotype data of European and East Asian ancestry respectively from population-matched 1000 Genomes Project data were simulated.<br><br>Sample size of the GWAS summary statistics: UKB (N = 314,921 - 360,388), BBJ (N = 42,790 — 159,095), PAGE(N = 11,178 Lipids Genetics Consortium (GLGC, N = 95,454 — 100,184), Genetic Epidemiology Network (AGEN, N = 27,657 — 34,374).<br><br>Sample size of the individual-level data: 2,683 East Asians and 763 admixed Americans in UKB after sample QC.
Data exclusions	Individual-level data: The full description of the quality control of the UKB data can be found in Methods in our manuscript. We removed samples already included in the UKB European GWAS. We also used KING to infer sample relatedness, and only kept individuals without any relatives at the third-degree or higher. We further excluded individuals with conflicting genetically-inferred and self-reported sex.<br><br>Summary statistics: no data exclusion.
Replication	We replicated our local genetic correlation results for four lipid traits using in independent data. We used European GWAS from the Global Lipids Genetics Consortium (GLGC, N = 95,454 — 100,184) and East Asian GWAS from the Asian Genetic Epidemiology Network (AGEN, N = 27,657 — 34,374) as the replication datasets.<br><br>We first consider uses the PRS to predict the East Asian phenotype. We demonstrated the improved PRS accuracy over all three other prediction tools. Then We replicated our improved PRS accuracy results by doing predicting to admixed American samples. We further replicated our improved PRS accuracy results on 31 traits in East Asian and 13 traits in admixed American. The averaged increase of R2 is 18.7%-122.1% compared to state-of-the-art methods based on GWAS summary statistics. Therefore, our results were replicated many times (i.e. for two ancestry population, 31 + 13 = 44 traits in total, and 3 other prediction tools).
Randomization	Simulations: the phenotype and genotype for individuals is randomly generated.<br><br>UKB PRS test sample analysis: The GWAS summary statistics were directly obtained from peer-reviewed publications. We randomly assign individuals from validation and test set. The summary statistics-based repeating learning is done by random splitting.<br><br>No other experiments were performed except for the two mentioned above.
Blinding	Blinding is not relevant to our study because we don't compare any case/control groups.

Reporting for specific materials, systems and methods

We require information from authors about some types of materials, experimental systems and methods used in many studies. Here, indicate whether each material, system or method listed is relevant to your study. If you are not sure if a list item applies to your research, read the appropriate section before selecting a response.

Materials & experimental systems

n/a	Involved in the study	
☒	☐	Antibodies
☒	☐	Eukaryotic cell lines
☒	☐	Palaeontology and archaeology
☒	☐	Animals and other organisms
☒	☐	Clinical data
☒	☐	Dual use research of concern

Methods

n/a	Involved in the study	
☒	☐	ChIP-seq
☒	☐	Flow cytometry
☒	☐	MRI-based neuroimaging
